# Subjective probability is modulated by emotions

**DOI:** 10.1038/s41598-025-92230-2

**Published:** 2025-03-14

**Authors:** Lara Abel, Eric Schulz, Jonathan D. Nelson

**Affiliations:** 1 School of Psychology, University of Surrey, Guildford, UK; 2 Research Strategy Office, University of Cambridge, Cambridge, UK; 3 MPRG Computational Principles of Intelligence, Max Planck Institute for Biological Cybernetics, Tübingen, Germany

**Keywords:** Probabilistic cognition, Emotion theories, Risk estimates, Emotional dominance, Conservatism, Subjective probability, Representativeness heuristic, Psychology, Human behaviour

## Abstract

Information about risks and probabilities is ubiquitous in our environment, forming the basis for decisions in an uncertain world. Emotions are known to modulate subjective probability when probabilistic information is desired (as in gambles) or undesired (as in risks). Yet little is known about the role of emotions in shaping the subjective probability of affectively neutral events. We investigated this in one correlational study (Study 1, *N* = 162) and one experimental study (Study 2, *N* = 119). As predicted, we found that participants higher in emotional dominance were more conservative in their probability estimates, avoiding the extremes. Remarkably, this pattern also transferred to realistic risk assessments. Furthermore, respondents’ tendency to use the representativeness heuristic as a proxy for probability was increased in high dominance individuals. Our findings suggest that emotional dominance may be a unifying construct explaining previously reported effects of emotions on probabilistic cognition.

## Introduction

Choices in financial investments, health, and even personal relationships require making decisions based on probabilistic information. Thus, a great deal of research has investigated how people estimate probabilities. The finding that people do not strictly follow the axioms of probability when evaluating probabilistic information was first observed in early work within the intuitive statistician framework^1,2^, and became famous with research on heuristics and biases^3,4^ and fast-and-frugal heuristics^5,6^.

Of key relevance in this context are compound events, also known as conjunctive events. Compound events are probabilistic events composed of a series of elementary events, each of which having a particular probability. In the classic Linda problem^7^, *Linda is a bank teller and active in the feminist movement* is a compound event, comprised of the elementary events *Linda is a bank teller* and *Linda is active in the feminist movement*. Generally speaking, the probability of a compound event is mathematically derived by applying the conjunction rule

1 $$\begin{aligned} P(A \, \text {and} \, B)= P(A|B) P(B) = P(B|A)P(A). \end{aligned}$$

In the case of independence of elementary events, this reduces to

2 $$\begin{aligned} P(A \, \text {and} \, B) = P(A) P(B). \end{aligned}$$

However, people often do not seem to follow this multiplicative approach. Instead, they typically tend to overestimate the probability of compound events^7–10^. More precisely, they avoid extreme probability estimates, especially if the true probabilities are close to zero^11,12^, and overestimate the probability of samples which appear representative of their population or the generating process^7,13–15^.

The tendency to overestimate small and underestimate large compound probabilities (albeit the latter tends to be less extreme), is also known as conservatism^2^. In the revision-of-opinion literature, conservative probability judgments result from a strong weight put on prior probabilities or background information, as opposed to likelihoods or individuating information, in a sequential sampling and belief updating process^2^. In the context of single compound probability estimates (where the probability of the *final result* of a sequential sampling process is estimated), conservatism describes the tendency to avoid extreme probability estimates, especially if the true probabilities are close to zero^11,12^. This results in more similar probability estimates for high and low probability compound events than is mathematically correct, that is, an overestimation of small and and an underestimation of large compound probabilities.

A number of models have been proposed to explain conservatism in probability estimates. Within a Bayesian framework, as in the Bayesian Sampler Model^16^, probability estimates can be seen as the result of a sampling process from memory with Bayesian updating using a generic prior. This prior can be thought of as a person’s initial beliefs, which are updated in the light of incoming evidence. Differences in people’s priors affect the degree of conservatism in their compound probability estimates: the stronger the prior, the more people tend to avoid the extremes, because incoming information has a smaller impact on the posterior probability in the updating process. Another way to explain conservatism is by assuming noise in human information processing, resulting in a regression of probability estimates toward a mean of 0.5^12^. This idea is also at the heart of the Probability Theory Plus Noise model (PT$+$N)^17^ which conceptualizes probabilistic cognition as fundamentally based on the axioms of probability theory. Deviations, such as conservatism, are explained by noise which is added to the representation of probabilistic information.

Another commonly found deviation from probability theory when assessing compound probabilities is a tendency to overestimate the relative probability of representative compound events^7,13^. In the Linda problem, Linda is described as a politically active, single, outspoken, bright woman. A person basing her judgment on representativeness would mistakenly assume that Linda being a bank teller *and* active in the feminist movement is more probable than Linda being a bank teller alone. A number of attempts to explain and define representativeness of a sample with respect to a population have been proposed in the literature. An idea which is intuitively appealing yet lacks a clear formalization is that representativeness is determined by the similarity between an observation and other observations resulting from the same generative process^14,15^. This line of argumentation is also at the heart of of Kahneman and Tversky’s^7,13^ conceptualization of representativeness as the correspondence between a sample and a population with respect to its essential properties^15^. Tenenbaum and Griffiths^15^ offer a Bayesian analysis of representativeness and propose a quantitative model of representativeness as diagnosticity (the log likelihood ratio as a measure of the goodness of a sample as an example of some generating process).

Considerations about the basic cognitive building blocks and processes underlying conservatism and representativeness judgments raise questions about inter- and intraindividual variability in subjective probability. A key source of inter- and intraindividual variability in cognition and behavior are emotions^18–35^. What is known about the role of emotions in subjective probability? Previous research has primarily focused on information environments in which probabilistic information was associated with positive or negative outcomes^24–28^. For these affectively valenced stimuli, positive emotions have been found to foster optimistic assessments, i.e., increased estimates for desired and decreased estimates for undesired outcomes, and negative emotions to foster pessimistic assessments, i.e., decreased estimates for desired and increased estimates for undesired outcomes^24,32^. Positive emotions have also been found to promote heuristic information processing, while negative emotions have been found to promote systematic processing of information^29–31^. One interpretation is that a positive emotion signals a safe environment and no need to engage in costly information processing, whereas a negative emotion signals a threat or a problem that requires a systematic analysis of the situation. These results support the view of an ecologically adaptive function of emotions as indicating to a decision maker when a risky decision is appropriate and when it is not^33,34^.

But emotions do not differ only in their valence. Cognitive emotion theories explain emotion-specific cognition as the result of activation patterns on several appraisal dimensions, such as uncertainty and control^19–23^. These in turn modulate the perception and evaluation of the environment. For instance, fear and anger, which are both negatively-valenced, are associated with very different appraisal patterns. Fear is characterized by high uncertainty and low control, and fosters systematic processing and pessimistic risk assessments. Anger is characterized by low uncertainty and high control, and fosters heuristic processing and optimistic risk assessments^20,28^.

Despite this rich theoretical framework for considering emotion and cognition, little is known about the role of emotions when estimating probabilities of affectively neutral events. Given the evidence that emotions interact with cognitive processes on a fundamental level, this gap in the literature is remarkable. Furthermore, from the literature it is not clear whether previously reported emotion-dependent probabilistic cognition may be attributed to the emotional content of the probabilistic information (e.g., emotion-congruent memory retrieval) *or* to a more general effect of emotions on probabilistic cognition. In other words, it is an open question whether emotions shape subjective probability on a fundamental level, regardless of the affective content of the probabilistic information. Our research sheds light on the little-understood interplay between emotions and estimates of neutral compound probabilities. More specifically, we investigated how emotions modulate two of the commonly found biases in probability judgments: conservatism, i.e., the avoidance of extreme probability estimates, and representativeness judgments, i.e., the relative overestimation of samples which are representative (or, in Bayesian terms, diagnostic) of an underlying generating process.

**Fig. 1:**
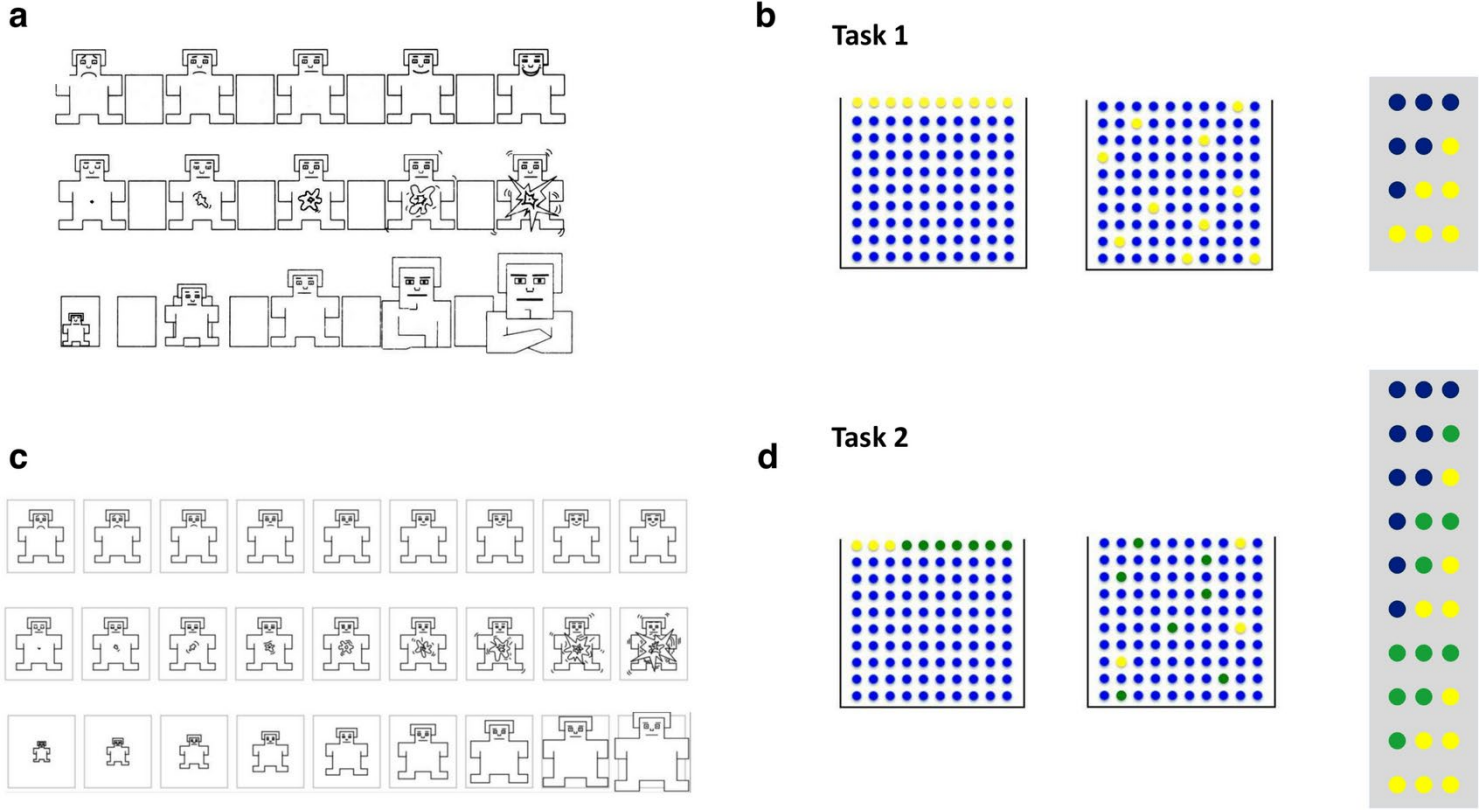
Material used in studies 1 and 2. (**a**) Self-Assessment-Manikin Scales from Bradley and Lang^38^ and Lang^42^. (**b**) Probability Task 1: Ordered distribution (90 blue, 10 yellow), mixed distribution (90 blue, 10 yellow), queried compound events (BBB, BBY, BYY, YYY). (**c**) Self-Assessment-Manikin Scales from PXLab^43^. (**d**) Probability Task 2: Ordered distribution (90 blue, 7 green, 3 yellow), mixed distribution (90 blue, 7 green, 3 yellow), queried compound events (BBB, BBG, BBY, BGG, BGY, BYY, GGG, GGY, GYY, YYY).

Study 1, which was preregistered, used a correlational design to find associative patterns between different characteristics of emotions and subjective probability, allowing a comparison of the relative predictiveness of different theories of emotions. This study was conducted at the onset of the COVID-19 pandemic and we expected participants to experience elevated levels of negative emotions, high uncertainty and low control associated with the pandemic. We were interested in the modulating role of these naturally occurring emotions and appraisal patterns in people’s probability estimates. In two tasks (Fig. 1b, d) participants estimated the probability of a series of compound events varying in mathematical probability that were generated from a known probability distribution. In a pretest, we assessed emotions (Fig. 1a) and cognitive appraisal. We found that emotional dominance predicted both conservatism and representativeness judgments in our compound probability estimation task. Emotional dominance is, according to the three-dimensional emotion model^36,37^, one dimension defining an emotion. It is characterized by a person’s perceived level of control, influence, autonomy^37,38^. Similar to cognitive appraisal of certainty and control, it can be used to distinguish emotions of the same valence, such as anxiety and anger^39^. Participants reporting higher levels of emotional dominance made increased use of the representativeness heuristic when estimating the relative probability of compound events, and their probability estimates were more conservative, avoiding the extremes.

In Study 2, we sought to experimentally replicate our findings from Study 1. This study, also preregistered, was conducted about one year after the onset of the COVID-19 pandemic. We experimentally induced emotional dominance using a subjective writing task, and asked participants to estimate the probability of a selection of the compound events from Study 1 (Fig. 1b). The findings from this study confirmed our hypothesis that people in a high dominance emotional state tended to be more conservative in their probability judgments and made increased use of representativeness as a proxy for probability.

As such, our work provides novel evidence that emotions not only affect probability estimates when outcomes are desired (as in gambles) or undesired (as in risks) but also when probabilistic events are affectively neutral. Whereas the emotion dimension valence has received a lot of attention in cognition-emotion research, the role of emotional dominance is not well understood. Based on our findings we propose emotional dominance, which is positively associated with emotional valence and characterized by a person’s perceived level of control, importance, autonomy, and influence, as a conceptual link between cognitive and valence-based theories of emotion and cognition.

## Methods

### Participants

In Study 1, *N* = 164 undergraduate psychology students from the University of Surrey and participants recruited on social media and research networks participated, out of which 162 produced valid data entries ($M_{\textit{age}}$ = 25.23, $SD_{age}$ = 15.27, range 18-88, 127 female, 33 male, 2 preferred not to say). Study 2 had *N* = 121 participants, with *N* = 119 complete and usable data files (*N* = 38 master’s conversion psychology students and *N* = 81 undergraduate psychology students from the University of Surrey, $M_{age}$ = 21.75, $SD_{age}$ = 5.93, range 18-49, 99 female, 18 male, 1 queer, 1 preferred not to say). For Study 2, a required sample size of 112 was determined using G*power^40,41^, for the test family *F-test*, statistical test *ANOVA: Repeated measures, within-between interaction*, a small effect size (0.02), an $\alpha$-level of 0.05 and a power of 0.8. Both studies were conducted fully online via Qualtrics and SoSciSurvey software. Participation was voluntary without reimbursement. Study 2 was conducted in an online Zoom call as part of a cognitive psychology course. Participants completed the study tasks independently but stayed in the joint Zoom call throughout the study. In Study 1, the same online Zoom setting was used for data collection with undergraduate psychology students. An exclusion criterion for participation in Study 1 was a previous COVID-19 infection. Both studies were conducted in accordance with University of Surrey ethical guidelines and the Declaration of Helsinki. The experimental protocols were approved by the ethical committee of the University of Surrey (ethical statement numbers 514292-514283-57083141 for Study 1 and 640816-640807-72456819 for Study 2). Written informed consent was obtained from all participants.

### Procedure

In Study 1, participants first gave written informed consent online. Then they rated their anticipated risk of a COVID-19 infection over the course of a year, completed a brief COVID-19 related appraisal questionnaire and indicated the anticipated consequences of an infection and whether they belonged to a risk group. Subsequently, participants rated their usual (trait) and current (state) emotional arousal, dominance and valence on the Self-Assessment-Manikin Scales^38^. Next, they completed two probability tasks, where they were asked to give probability estimates for drawing several three item color combinations with replacement from jars filled with 100 balls of two (Task 1) and three (Task 2) different colors (Fig. 1b, d). Finally, demographic data (age, gender, country of residence) were collected. In Study 2, participants first gave informed consent online. They rated their usual (trait) and current (state) emotional dominance, valence and arousal on nine-point Self-Assessment-Manikin^38^ scales. Participants were randomly assigned to either the high or low dominance emotion condition and completed a subjective writing task to induce high or low dominance, as described below. After the emotion induction, participants indicated their subjective risk of an infection with COVID-19 over the course of the following year, and completed probability Task 1 from Study 1. Finally, they completed the same COVID-19 related questions as in Study 1, except for the appraisal questionnaire, and additionally indicated their vaccination status, and their self-rated mathematics and statistics proficiency. In both studies, participants completed the questionnaires at their own pace and online (Qualtrics).

### Materials

#### Covid-19 questions

Participants rated their subjective Covid-19 infection risk (in percent) in the next three days, week, month, three months and year using a slider, the perceived consequences of an infection (on a five-point Likert scale), whether they belonged to a risk group, and their COVID-19 vaccination status (in Study 2 only). In Study 1, participants also rated their subjective appraisal of current and future uncertainty, control, mastery and anxiety with respect to the COVID-19 pandemic on seven-point Likert scales. See Supplementary Methods S1 for wording of items.

#### Emotion measure

Emotional valence, arousal and dominance were assessed using the nine-point version of the Self-Assessment-Manikin scales^38,42^. Instructions were based on material provided by the authors, see Supplementary Methods S2. In Study 1, visual stimuli were taken from the PXLab website of the University of Mannheim^43^. For these stimuli, each answer option corresponds to one particular manikin, thus the last sentence of the instructions (*You can select any answer option between the extremes to indicate intermediate levels of emotional dominance*) was deleted. In Study 2, visual stimuli by Lang (1980)^42^ were used (Fig. 1c).

#### Emotion induction

In Study 2, high and low emotional dominance was induced using a subjective writing task^28,44^. First, participants were asked to list five autobiographical or imagined situations in which they would feel a certain way and describe them in 2-3 sentences each. Then they were asked to write about one of these situations in more detail (using five sentences) so that a person reading it would feel the same way. In the high dominance condition, participants were instructed to write about situations in which they would feel (1) *angry*, (2) *furious and outraged*, (3) *in control and dominant*, (4) *strong and autonomous* and (5) *influential and important*. In the low dominance condition, participants were instructed to write about situations in which they would feel (1) *anxious*, (2) *scared*, (3) *controlled and influenced*, (4) *weak and cared for* and (5) *awed, submissive and unimportant*. In the first lab session (*N* = 37 students), instructions slightly differed from this method: subtasks (3)–(5) were grouped and participants were asked to write about this emotion-eliciting event in more detail. The groups did not differ in induced emotional dominance between data collection time points (both when analyzing change scores and emotional dominance after the emotion induction), thus data were pooled.

#### Probability task

In Study 1, participants completed two probability tasks. In Probability Task 1, we showed participants an image of a jar containing 100 balls, 90 of which were blue and 10 yellow, first ordered by color, and then an example image of a mixed jar. In a first step, we asked participants which of four color combinations would most likely be sampled, in random order: BBB, BBY, BYY, or YYY. Next, participants were asked to estimate the probability of each of the four color combinations, again in random order. In Study 2, we adapted the instructions to make it more explicit that the queried color combinations referred exactly to the displayed combinations in the picture. We specified: *note that blue-blue-yellow represents drawing a blue ball in the first and second draw and a yellow ball in the third draw; blue-yellow-yellow represents drawing a blue ball in the first draw and a yellow ball in the second and third draw.* Participants were asked to estimate the probability in percent of drawing each of the four color combinations on a slider with values ranging from 0 to 100 (increments of 1 in Study 1 and increments of 0.235 in Study 2). Task 2 in Study 1 was similar to Task 1, except that the constitutive jar contained 100 balls of three different colors: 90 blue, 7 green, and 3 yellow balls. Consequently, participants rated 10 different color combinations. Instructions can be found in Supplementary Methods S4.

### Statistical methods

Data were analyzed using R, version 4.0.2. Linear mixed models were fitted using the *lmer()* function from the *lme4* package^45^. Analyses were validated using the *robustlmm* package (version 2.4.4)^46^. In all mixed models, participants were included as random effects. $R^2$ was calculated using the *r.squaredGLMM()* function from the *MuMIn* package (version 1.43.17)^47^, returning marginal effects (relative variance explained by fixed effects) and conditional effects (variance explained by the complete model including fixed and random effects). Predictor variables were z-standardized before performing regressions. Bootstrapped 95% confidence intervals (*CIs*) were obtained using the *confint.merMod()* function from the *lme4* package. $\beta$ coefficients and $\sigma$ values were bootstrapped with 10000 simulations per model. Type III ANOVAs using Satterthwaite’s method were run on each model to obtain *F*-statistics and *p*-values. Logistic regressions were fitted using the *glm()* method with the argument *family = binomial(“logit”)* and model parameters bootstrapped using the *boot()* function from the *boot* package (version 1.3.28)^48^ with 10000 simulations for each test. Plots were generated the *ggplot2* (Version 3.3.5), *gghalves* (Version 0.1.1), *ggtext* (Version 0.1.1 ), *gridExtra* (Version 2.3), *png* (Version 0.1-7), and *cowplot* (Version 1.1.1) packages.

## Results

### Study 1

We asked participants to rate their current (state) and usual (trait) emotional valence, dominance and arousal, their anxiety and cognitive appraisal of uncertainty regarding the current situation and future developments, as well as subjective control associated with COVID-19, in the early stages of the pandemic (March and April 2020). In two tasks, participants estimated the probabilities of neutral compound events, i.e., the probabilities of sampling various three-item color combinations from known probability distributions (Fig. 1b, d; e.g., BBB, BBY, BYY and YYY in Task 1, where B stands for the color blue and Y stands for the color yellow). Participants also rated the expected risk of a COVID-19 infection for time intervals up to one year in the future. The data sets as well as the code for statistical analyses are available on the Open Science Framework, as specified in the Data Availability Statement.

Participants reported lower average levels of emotional valence and dominance at the onset of the pandemic compared to their usual valence and dominance. Ratings of uncertainty and anxiety were generally high, and ratings of subjective control low. In both probability tasks, participants systematically overestimated the probability of compound events, except for the compound event containing only the most probable kind of item (BBB). This overestimation was more pronounced in individuals reporting high emotional dominance. In comparison to participants low in dominance, high-dominance individuals gave higher estimates for low probability compound events and lower estimates for high probability compound events. In other words, probability estimates of participants reporting high emotional dominance differed relatively little between high and low probability events. This effect can be interpreted as greater conservatism in high-dominance participants.

Was there also a relationship between dominance and representativeness? The most representative compound event (Kahneman and Tversky, 1972, p. 430) is the one that is most “similar in essential characteristics to its parent distribution”^13^. In our task, the key properties of the BBY compound event are that it contains the colors of the generating jar, and that the most frequent color in the underlying jar is the most frequent color in the sample. By contrast, BBB, despite being mathematically more probable, does not contain both colors, and is hence less representative than BBY. When asked to select the combination with the highest probability of being sampled, individuals higher in dominance showed a greater tendency to selected the most representative event over the mathematically most probable compound event.

#### Data preprocessing and statistical methods

Before analyzing participants’ probability estimates, we tested whether belonging to a COVID-19 risk group and anticipated personal consequences of a COVID-19 infection predicted participants’ emotional state and their subjective infection risk estimates. Out of 162 participants, 24 indicated that they belonged to a risk group, 15 did not report whether they belonged to a risk group or not, and 123 indicated that they did not belong to a risk group. A MANOVA with risk group categorization and self-rated infection consequences as predictors of state valence, dominance and arousal showed that belonging to a risk group ($\text {Pillai's trace} = 0.06, F\text {(6, 314)} = 1.75, p = 0.11$) and anticipated personal consequences of an infection ($\text {Pillai's trace} = 0.02, F\text {(3, 156)} = 0.99, p = 0.40$) did not significantly predict participants’ emotional state. Neither did belonging to a risk group ($\text {Pillai's trace} = 0.07, F\text {(10, 280)} = 1.02, p = 0.42$) nor anticipated personal consequences of an infection ($\text {Pillai's trace} = 0.06, F\text {(5, 139)} = 1.69, p = 0.14$) affect participants’ infection risk estimates. Thus, these variables were not included as controls in subsequent analyses.

Unless explicitly stated, the predictors in our analyses were participants’ scaled raw responses on Likert scale variables. *Only* for visual presentation of results from mixed models (but not for analyses), participants were categorized according to the level of their reported emotional valence and dominance, so as to obtain roughly equally sized groups. Categories were *low* (a value below the median), *median* (exactly the median) and *high* (above the median). Binary categories were created to visualize the self-reported effect of the COVID-19 pandemic on participants’ emotional state: *increasing*, meaning that the difference between usual/trait and current/state emotional valence/dominance was positive or zero, and *decreasing*, meaning that the difference between usual/trait and current/state emotional valence/dominance was negative.

We also applied a logit transformation to participants’ probability estimates. Logit-transformed values indicate how extreme probability estimates are, and thus provide a more direct index of conservatism than can be seen in raw probability estimates^14,49^. The further a transformed value is from 0 (which is the log-odds of 50% or 0.5), the more extreme the probability estimate. Values closer to zero indicate greater conservatism, i.e., reluctance to give extreme probability estimates. To avoid having infinite values in the analyses, and because the closest value participants could select on the slider to approximate the mathematically correct probability for compound event YYY (0.001) was 0, probability estimates of 0% were replaced by 0.001, and estimates of 100% were replaced by 99.999, resulting in log odds of $-5$ and 5, respectively. All analyses were conducted for both raw probability estimates and for logit-transformed values. Results were very comparable for the two types of analyses, although results for the logit-transformed values tended to be stronger. Because the raw probability estimates are easiest to interpret in reference to the probability task, we report those analyses here. Visualisations of analyses using logit-transformed data can be found in Supplementary Figures S3 and S4.

**Fig. 2:**
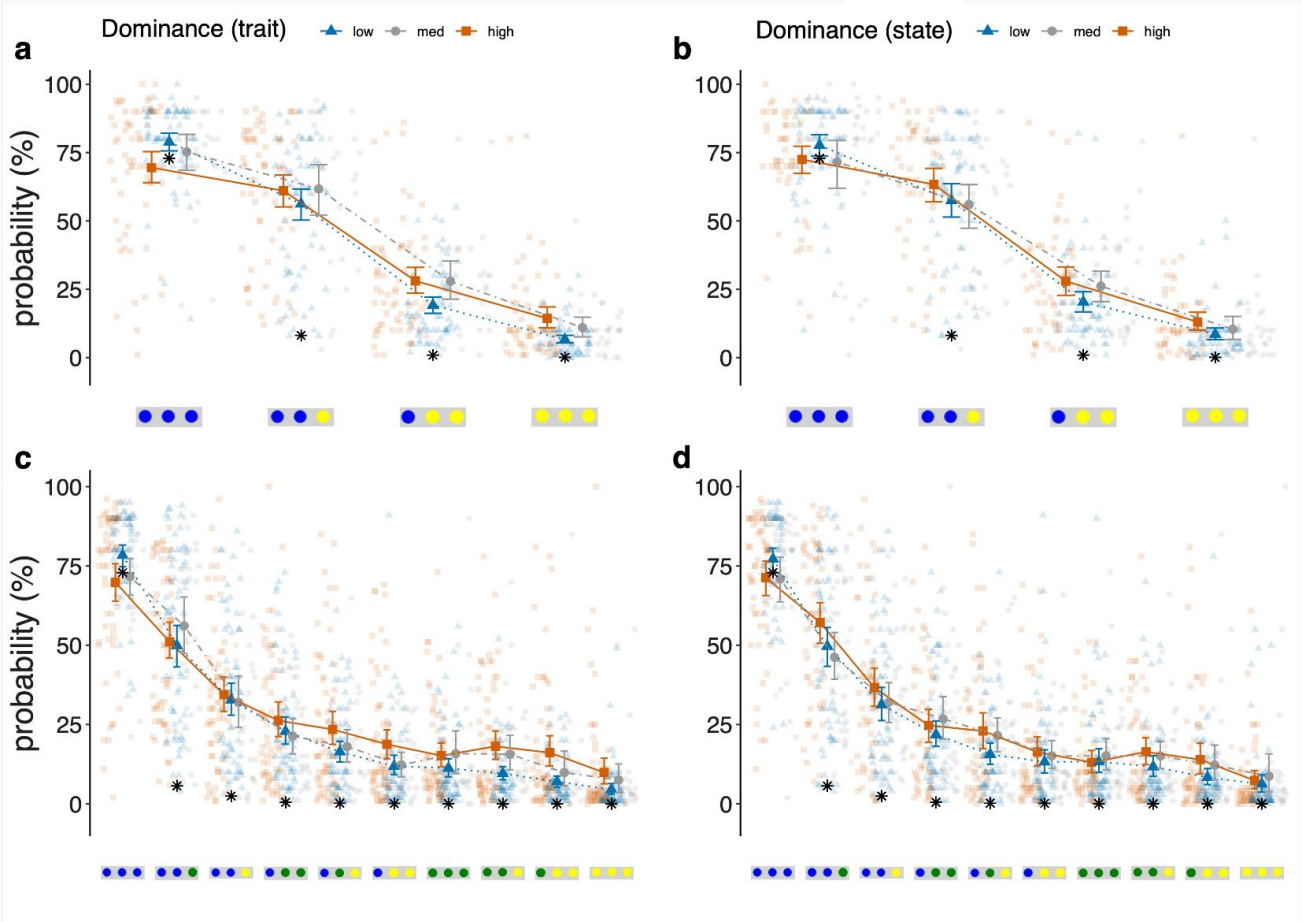
The plots display participants’ probability estimates by emotional dominance in Study 1 for different compound events in Task 1 (**a**, **b**) and Task 2 (**c**, **d**). Queried compound events are plotted on the x-axis, ordered by magnitude of true mathematical probability (decreasing from left to right). The y-axis displays probability estimates in percent. True probabilities are indicated as black asterisks. Categories for emotional dominance are: low = values below the median; median = values exactly at the median; high = values above the median. Groups had approximately the same size. (**a**), (**b**) Compound probability estimates for Task 1. (**c**), (**d**) Compound probability estimates for Task 2. (**a**), (**c**) Participants’ probability estimates by usual (trait) dominance before the onset of the Covid-19 pandemic (retrospectively reported). 95% *CI*s are displayed for each group. (**b**), (**d**) Participants’ probability estimates by self-reported state dominance at the onset of the Covid-19 pandemic. 95% CIs are displayed for each group.

#### Emotional dominance and conservatism

In a first step, we tested whether age and gender predicted participants’ probability estimates. For this, we fitted linear mixed models predicting participants’ probability estimates by *queried compound events* (4 queried events in Task 1, 10 in Task 2), age and gender. Age ($\textit{F}(1, 156) = 4.84$, $\textit{p} = 0.03$) and gender ($\textit{F}(2, 156) = 3.56$, $\textit{p} = 0.03$) explained variance in participants’ probability estimates in Task 1 but not in Task 2 (both *p* > 0.05). Thus, we included age and gender as control variables in our analyses of probability estimates in Task 1.

Next, we explored the relationship between emotions and conservatism in participants’ estimates of the neutral compound probabilities in Tasks 1 and 2. Increased conservatism would mean that a person gives relatively high estimates for low probability and relatively low estimates for high probability compound events, avoiding the extremes. For each of the two task, we fitted separate linear mixed models with respondents’ *probability estimates* as the dependent variable, *queried compound events* as within subjects repeated measures variable and the between-subjects variables a) *state valence*, *state dominance* and *state arousal*, and b) *trait valence*, *trait dominance* and *trait arousal*. We included all three emotion dimensions to derive incremental variance explained by emotional dominance. We controlled for age and gender in all mixed models predicting probability estimates in Task 1. Across tasks, *state dominance* and *trait dominance* explained a significant part of the variance in participants’ probability estimates (Fig. 2).

Note that we conducted all analyses with raw and logit-transformed values to confirm robustness of our findings. Results were very similar for both kinds of analyses. Here we report results for the more easily interpretable raw probability estimates. Visualizations of our analyses using logit-transformed values can be found in Supplementary Figure S3.

We fitted a linear mixed model predicting probability estimates in Task 1 by *queried compound event* (within-subjects variable), *state valence*, *state dominance* and *state arousal* (between-subjects variables), and *age* and *gender* (control variables). We found a main effect of *queried compound event* ($\textit{F}(3, 468) = 471.22$, $\textit{p} < 0.0001$) and an interaction between *state dominance* and *queried compound event* ($\textit{F}(3, 468) = 3.03$, $\textit{p} = 0.03$). Age ($\textit{F}(1, 153) = 4.03$, $\textit{p} = 0.05$) and gender ($\textit{F}(2, 153) = 3.61$, $\textit{p} = 0.03$) also predicted variance in participants’ probability estimates. Overall, this model explained 71.22 % of the variance in the data (65.72 % by fixed effects alone). Participants reporting higher state dominance gave lower estimates for the high probability compound event BBB, and they gave higher probability estimates for the low probability compound events BBY and BYY than participants scoring low on dominance. This indicates that participants feeling high in dominance avoided the extremes and gave more similar probability estimates, i.e., showed greater conservatism, than participants low in dominance. Bootstrapped coefficients of the model can be found in Table 1. Results are visualized in the top right plot in Fig. 2.

**Table 1:** Bootstrapped model coefficients and inferential statistics of the linear mixed model predicting participants’ probability estimates in Study 1, Task 1, by state emotions.

State emotions	Coefficients			Statistics	
	$\beta$	*SE*	95 % *CI*	*t*	*p*
Intercept	81.03	11.63	[58.470, 103.584]	6.93	< 0.0001***
bby	$-$ 15.43	1.97	[$-$ 19.192, $-$ 11.658]	$-$ 7.92	< 0.0001***
byy	$-$ 50.30	1.93	[$-$ 54.067, $-$ 46.533]	$-$ 25.93	< 0.0001***
yyy	$-$ 64.18	1.96	[$-$ 67.942, $-$ 60.408]	$-$ 33.07	< 0.0001***
dom:bby	4.44	2.07	[0.421, 8.458]	2.14	0.03*
dom:byy	5.71	2.08	[1.686, 9.724]	2.75	0.006**
dom:yyy	4.84	2.08	[0.820, 8.857]	2.34	0.02*
age	$-$ 1.90	0.95	[$-$ 3.723, $-$ 0.076]	$-$ 2.01	0.05*

Only coefficients significantly different from zero are listed.

A similar pattern, that is, a significant interaction between *emotional dominance* and *queried compound event* (*F*(3, 468) = 2.77, *p* = 0.04) emerged for *trait dominance* (top left plot in Fig. 2). In this model, which explained 72.07 % of the variance (66.72 % by fixed effects alone), we also found main effects of *queried compound event* ($\textit{F}(3, 468) = 485.79$, $\textit{p} < 0.0001$) and the control variables age ($\textit{F}(1, 153) = 6.65$, $\textit{p} = 0.01$) and gender ($\textit{F}(2, 153) = 3.03$, $\textit{p} = 0.05$), and an interaction between *queried compound event* and *trait valence* ($\textit{F}(3, 468) = 2.68$, $\textit{p} = 0.05$). Bootstrapped coefficients of the model can be found in Table 2. Results are visualized in the top left plot in Fig. 2.

**Table 2:** Bootstrapped model coefficients and inferential statistics of the linear mixed model predicting participants’ probability estimates in Study 1, Task 1, by trait emotions.

Trait emotions	Coefficients			Statistics	
	$\beta$	*SE*	95 % *CI*	*t*	*p*
Intercept	80.78	11.46	[58.550, 103.696]	7.03	< 0.0001***
bby	$-$ 15.43	1.94	[19.195, $-$ 11.560]	$-$ 8.06	< 0.0001***
byy	$-$ 50.30	1.90	[$-$ 53.977, $-$ 46.554]	$-$ 26.29	< 0.0001***
yyy	$-$ 64.18	1.93	[$-$ 67.918, $-$ 60.361]	$-$ 33.54	< 0.0001***
dom:byy	5.92	2.22	[1.583, 10.295]	2.67	0.008**
dom:yyy	4.76	2.22	[0.483, 9.181]	2.15	0.03*
val:bby	5.89	2.22	[1.490, 10.175]	2.68	0.008**
val:byy	4.50	2.19	[0.272, 8.803]	2.05	0.04*
age	$-$ 2.49	0.96	[$-$ 4.379, $-$ 0.564]	$-$ 2.58	0.01*

Only coefficients significantly different from zero are listed.

We had taken the COVID-19 pandemic as a naturally occurring manipulation of people’s emotions. Thus, we were interested in the relationship between the self-reported emotional effect of the pandemic (the difference between *state* and *trait* emotions), and participants’ probability estimates. In a linear mixed model predicting probability estimates by *queried compound event* and *emotional dominance*, *valence* and *arousal difference*, *valence difference* interacted with *queried compound event* (*F*(3, 468) = 2.85, *p* = 0.04) in predicting probability estimates. The control variables age (*F*(1, 153) = 4.54, *p* = 0.03) and gender (*F*(2, ) = 3.52, *p* = 0.03) also explained a significant part of the variance. Bootstrapped model coefficients can be found in Table 3. Participants reporting lower *state valence* than *trait valence*, that is, who reported being emotionally more negatively affected by the pandemic, were more likely to be conservative in their probability estimates. At first sight, this result contradicts previous research showing that negative emotional states foster systematic information processing^29,31,50^. However, one has to keep in mind that when looking at the *difference* between current (state) and usual (trait) emotional valence, we are considering trait and state emotional valence both at the same time. How exactly state and trait emotions interact has not fully been understood^51^. When analyzed separately, state and trait emotional valence did not explain a significant share of the variance in probability estimates. Given that our result is only on the boundary of significance, we are reluctant to make claims about the modulating role of state-trait emotion interactions in subjective probability at this point.

**Table 3:** Bootstrapped model coefficients and inferential statistics of the linear mixed model predicting participants’ probability estimates in Study 1, Task 1, by the difference between current (state) and usual (trait) emotions (only coefficients significantly different from zero are listed).

Difference in state and trait emotions	Coefficients			Statistics	
	$\beta$	*SE*	95 % *CI*	*t*	*p*
Intercept	83.21	11.62	[60.592, 106.339]	7.13	0.000***
bby	$-$ 15.43	1.97	[$-$ 19.257, $-$ 11.498]	$-$ 7.93	< 0.0001***
byy	$-$ 50.30	1.93	[$-$ 54.036, $-$ 46.493]	$-$ 25.87	< 0.0001***
yyy	$-$ 64.18	1.96	[$-$ 67.978, $-$ 60.300]	$-$ 33.00	< 0.0001***
val:bby	$-$ 4.07	2.07	[$-$ 8.148, $-$ 0.036]	$-$ 1.98	0.05*
val:byy	$-$ 4.79	2.06	[$-$ 8.756, $-$ 0.713]	$-$ 2.34	0.02*
val:yyy	$-$ 5.44	2.04	[$-$ 9.370, $-$ 1.395]	$-$ 2.65	0.008**
age	$-$ 2.23	1.05	[$-$ 4.290, $-$ 0.184]	$-$ 2.13	0.03*

We ran the same analyses for probability estimates in Task 2 (Fig. 2c, d) and replicated most of our results. As in Task 1, we found main effects of *queried compound event* and interactions between *queried compound event* and *state* ($\textit{F}(9, 1422) = 2.35$, $\textit{p}=0.01$) and *trait dominance* ($\textit{F}(9, 1422) = 2.29$, $\textit{p} = 0.02$). Gender ($F(2, 156) = 2.15, p = 0.12$) and age ($F(1, 156) = 1.52, p = 0.22$) were did not significantly predict probability estimates in Task 2 and were therefore not included in the linear models predicting probability estimates in Task 2 by emotions. Individuals high in *state dominance* gave relatively low estimates for the item BBB but relatively high estimates for all other queried items (bootstrapped *B* between 4.39 and 7.04, *SE B* between 1.73 and 1.76). In this model, we also found a main effect of *dominance* on probability estimates (bootstrapped $\textit{B} = -3.13$, 95% *CI* $[-6.06, -0.18]$, $\textit{SE B} = 1.51$, $p = 0.04$. With increasing *state dominance*, individuals tended to give lower estimates for the high probability and higher estimates for all other compound events, overestimating the probability of the low probability events. Effectively, they tended to give more similar answers for the different compound events (Fig. 2d). A similar pattern emerged for *trait dominance*. Within the model predicting probability estimates by trait emotions and compound event, we found interaction effects of *trait dominance* and *queried compound event* on estimates for all other compound events (bootstrapped *B* between 2.39 for BBG, and 6.63 for GYY, *SE B* between 1.90 and 1.93), except for the item BBG. This means that the modulating role of dominance in probability estimates differed significantly between the high probability item BBB and all other items, except for BBG: For the high probability compound event BBB participants higher in dominance gave lower estimates and for the low likelihood compound events they gave higher estimates than participants scoring lower on dominance. Neither *valence* nor *dominance* or *arousal difference* predicted probability estimates in Task 2 (all $\textit{p} > 0.05$).

**Fig. 3:**
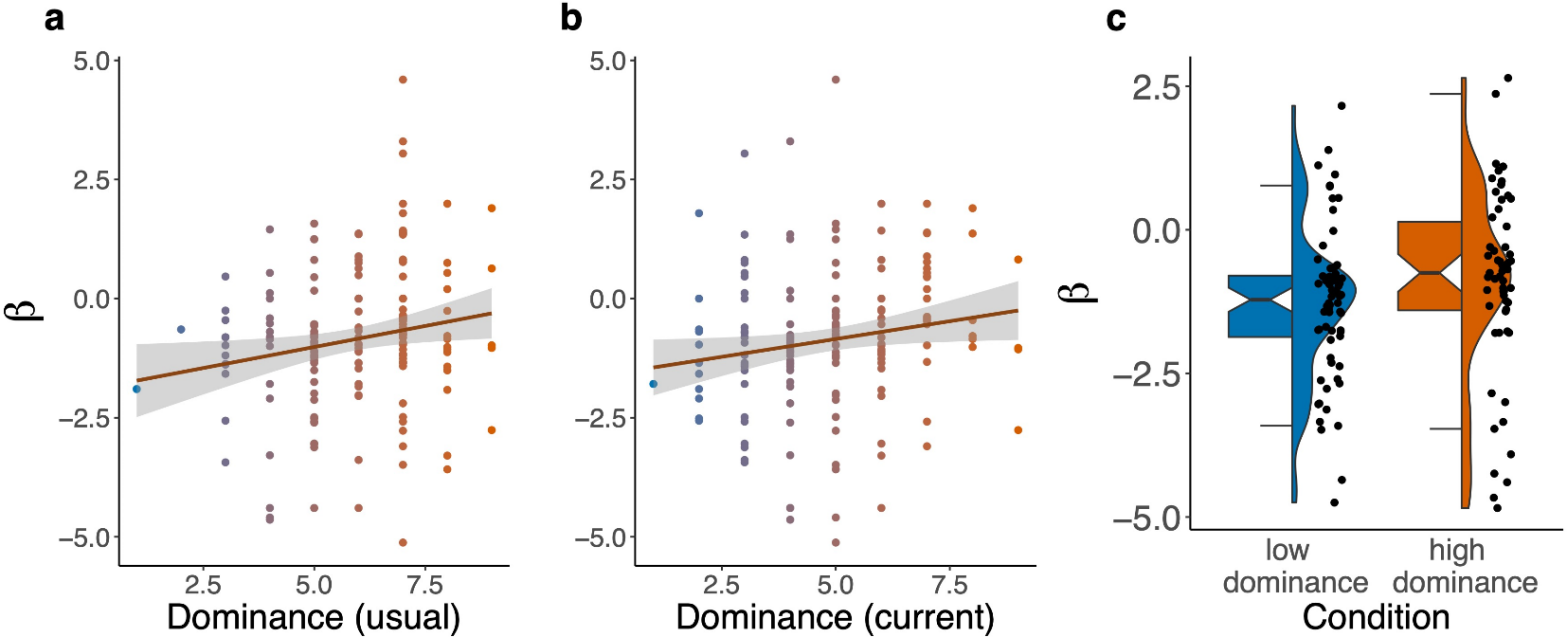
Visualization of the relationship between participants’ conservatism and emotional dominance in Studies 1 and 2. (**a**) Scatterplot and regression line with standard error for the relationship between participants’ self-reported trait dominance and regression coefficients extracted from the model predicting logit-transformed probability estimates by *queried compound event* in Study 1. (**b**) Scatterplot and regression line with standard error for the relationship between participants’ self-reported state dominance and regression coefficients extracted from the model predicting logit-transformed probability estimates by *queried compound event* in Study 1. (**c**) Effect of the emotion manipulation (high vs. low dominance) on regression coefficients extracted from the model predicting logit-transformed probability estimates by *queried compound event* in Study 2. The plot shows notched boxplots, data distributions and individual data points in each experimental condition (left/green: low dominance, right/red: high dominance).

To test whether the association between emotional dominance and probability estimates also emerged on an individual level, we performed a Regression Coefficient Analysis (RCA). For this, we regressed the within-subjects variable *queried compound event* (compound events ordered by probability: BBB, BBY, BYY, YYY) on probability estimates (both raw values and logit-transformed values) for each participant individually. We included *age* and *gender* as control variables in these models. In a next step, we extracted participants’ individual regression coefficients. Each regression coefficient tracks how much a single participant reacted to differences in the mathematical probability of compound events when estimating their probabilities. In Bayesian terms, when assuming an uninformative prior, this coefficient tracks how much participants react to the likelihood of compound events. In a frequentist framework, it quantifies how much a participants’ probability estimates regress towards the mean. Because of the ordering of compound events from high to low probability, higher coefficients mean that people reacted less, estimating high and low probability compound events as relatively similar. That is, these participants avoided the extremes and showed greater conservatism. We then tested whether this participant-specific measure of conservatism was modulated by dominance by regressing *trait* and *state dominance* on this parameter. Indeed, people higher in trait dominance (Task 1: $F(1, 155) = 7.28, p = 0.008$; Task 2: $F(1, 155) = 4.31, p = 0.04$) and state dominance (Task 1: $F(1, 155) = 5.17, p = 0.02$; Task 2: $F(1, 155) = 5.25, p = 0.0005, R^2 = 0.10$) had smaller beta weights, which means they gave relatively similar probability estimates. They reacted less to the likelihood or, in frequentist terms, regressed their estimates more towards the mean. In these models, gender also explained a significant part of the variance (Task 1: $F(2, 155) = 6.86, p = 0.001$ when predicting trait dominance, $F(2, 155) = 7.04, p = 0.001$ when predicting state dominance; Task 2: $F(2, 155) = 6.14, p = 0.003$ when predicting trait dominance, $F(2, 155) = 6.32, p = 0.002$ when predicting state dominance). None of the bootstrapped coefficients for gender were significant in the linear model. A visualization of the results of these analyses can be found in Fig. 3. Bootstrapped model coefficients are listed in Table 4.

**Table 4:** Study 1: Results from Regression Coefficient Analyses. Bootstrapped model coefficients (*b*) and Confidence Intervals (*CI*) of the models predicting probability estimates in Task 1 (top) and Task 2 (bottom) by age, gender, and emotions.

	$b_{int}$	$CI_{int}$	$b_{dom}$	$CI_{dom}$	$b_{f}$	$CI_{f}$	$b_{m}$	*CI*
* Task 1*								
Dom S	$-$ 8.07	[$-$ 52.670, 36.39]	4.55	[0.409, 8.699]	$-$ 3.47	[$-$ 48.309, 41.364]	$-$ 23.23	[$-$ 68.918, 22.464]
Dom T	$-$ 8.89	[$-$ 53.223, 35.433]	5.55	[1.407, 9.688]	$-$ 2.64	[$-$ 47.198, 41.915]	$-$ 22.04	[$-$ 67.455, 23.378]
* Task 2*								
Dom S	$-$ 47.57	[$-$ 92.884, $-$ 2.252]	5.79	[1.584, 10.005]	28.79	[$-$ 16.763, 74.339]	10.30	[$-$ 36.124, 56.715]
Dom T	$-$ 48.06	[$-$ 93.868, $-$ 2.246]	4.37	[0.092, 8.651]	29.34	[$-$ 16.702, 75.392]	10.93	[$-$ 36.007, 57.866]

1000 samples were drawn. Only samples which included both genders were included in the computation of summary statistics. Note that wile the overall effect of gender was significant in all ANOVAs, individual coefficients for gender in the linear model were not significant. *int* stands for *Intercept*, *dom* stands for *emotional dominance*, *f* stands for *female* and *m* stands for *male*.

#### Emotional dominance and representativeness judgments

Emotion theories predict that emotions characterized by positive valence and high certainty and control foster heuristic information processing. Accordingly, we expected increased heuristic information processing in participants reporting high emotional dominance and valence relative to those reporting low emotional dominance and valence. A heuristic that has been reported in the literature on subjective probability assessments of compound events is the representativeness heuristic^8,13^. Did participants high in dominance and valence make greater use of representativeness in their probability estimates?

To test this, we ran logistic regressions of participants’ self-reported state and trait emotional dominance and valence on their choice for the most probable compound event in Task 1. More specifically, we contrasted choices for the compound event BBB (highest mathematical probability) and BBY (most representative). The results confirm a positive relationship between emotional dominance and valence and the choice of the subjectively most probable compound event. The higher a person’s emotional state and trait dominance, the more likely a person was to select the most representative compound event over the mathematically most probable compound event. Trait but not state valence predicted the selection of the most probable compound event, with participants reporting more positive trait valence being more likely to select the most representative compound event as most probable. We ran the same analyses for participants’ choices of the most probable compound event in Task 2 (contrasting choices for BBB and BBG). Effects were less pronounced but went in the same direction for dominance and were stronger for valence. Results can be found in Table 5.

**Table 5:** Study 1: Results from logistic regressions predicting participants’ selection of the subjectively most probable compound event by state and trait emotional dominance and valence.

	Coefficients			Statistics		
	$\beta$	*SE*	95 % *CI*	*p*	*OR*	95 % *CI*
* Task 1*						
State dominance	0.20	0.10	[0.016, 0.398]	0.04*	1.22	[1.016, 1.489]
Trait dominance	0.21	0.11	[0.000, 0.425]	0.05	1.23	[1.000, 1.530]
State valence	0.10	0.12	[$-$ 0.103, 0.302]	0.34	1.10	[0.903, 1.352]
Trait valence	0.29	0.12	[0.069, 0.527]	0.01*	1.33	[1.071, 1.693]
* Task 2*						
State dominance	0.20	0.11	[$-$ 0.004, 0.406]	0.06	1.22	[0.996, 1.501]
Trait dominance	0.18	0.12	[$-$ 0.034, 0.406]	0.11	1.20	[0.966, 1.501]
State valence	0.25	1.23	[0.035, 0.468]	0.03*	1.28	[1.036, 1.597]
Trait valence	0.33	0.12	[0.099, 0.585]	0.007**	1.39	[1.105, 1.795]

### Study 2

In Study 1, participants reporting high emotional dominance gave more similar probability estimates for high and low probability compound events relative to participants reporting low emotional dominance. In other words, the degree to which participants tended to be conservative and avoid extreme probability estimates was positively associated with dominance at both the group and individual level. Furthermore, participants high in emotional dominance and valence were more likely to choose as most probable a representative compound event (BBY in Task 1 and BBG in Task 2) over the compound event with the highest mathematical probability. However, the correlational nature of Study 1 does not allow any causal inference about the relationship between emotional dominance and probabilistic cognition.

In Study 2 we therefore replicated the key components of Study 1, using an experimental manipulation of emotional dominance. Participants were randomly assigned to one of two conditions in an autobiographical writing task designed to induce high or low emotional dominance. Results suggest a causal effect of emotional dominance on conservatism and representativeness judgments in neutral compound probability estimates.

#### Participant characteristics and manipulation check

Experimental groups did not differ in their self-reported proficiency in mathematics and statistics ($\textit{W} = 1741.5$, $\textit{p} = 0.92$, $\textit{d} = 0.01$); the proportion of participants with a previous COVID-19 infection (9.4% in the low dominance condition, 12.7% in the high dominance condition); gender distribution (15.9% males in the low dominance condition, 12.7% males in the high dominance condition); age ($\textit{W} = 1952.5$, $\textit{p} = 0.29$, $\textit{d} = 0.07$); trait dominance ($\textit{W} = 2047.5$, $\textit{p} = 0.12$, $\textit{d} = 0.1$) or proportion of participants vaccinated against COVID-19 (87.5% not vaccinated in the low dominance condition, 94.5% in the high dominance condition). Thus, these variables were not included as control variables in subsequent analyses.

To test the effectiveness of the emotion manipulation, we compared dominance, valence and arousal ratings after the emotion induction, as well as differences between ratings before and after the emotion induction (change scores) between conditions. After the emotion induction, emotional dominance was higher in the high dominance condition ($\textit{M}_{\textit{post}} = 5.6$, $\textit{SD} = 1.76$) than in the low dominance condition ($M_{\textit{post}} = 4.61$, $\textit{SD} = 1.82$; $\textit{W} = 2339.5$, $\textit{p} = 0.002$, $\textit{d} = 0.2$). Dominance change scores were computed by subtracting pre from post emotion induction scores. Dominance increased in the high dominance condition ($M_{\textit{change}} = 0.64$, $\textit{SD} = 1.98$) but did not in the low dominance condition ($M_{\textit{change}} = -0.1$, $\textit{SD} = 1.78$). This difference in change scores was significant ($\textit{W} = 2125.5$, $\textit{p} = 0.05$, $\textit{d} = 0.13$). Emotional valence was also higher in the high dominance condition ($M_{\textit{post}} = 5.49$, $\textit{SD} = 1.6$) than in the low dominance condition ($M_{\textit{post}} = 4.72$, $\textit{SD} = 1.77$; $\textit{W} = 2248$, $\textit{p} = 0.008$, $\textit{d}= 0.17$). The change in valence significantly differed between groups ($\textit{W} = 2276.5$, $\textit{p} = 0.005$, $\textit{d}= 0.18$): In the high dominance condition valence change was positive ($M_{\textit{change}} = 0.05$, $\textit{SD} = 1.89$) whereas in the low dominance condition it was negative ($M_{\textit{change}} = -0.64$, $\textit{SD} = 1.42$). Experimental groups did not differ in emotional arousal after the emotion induction ($\textit{W} = 1874$, $\textit{p} = 0.54$) or in arousal change ($\textit{W} = 1926$, $\textit{p} = 0.37$). These findings strongly suggest that the emotion manipulation was successful. They also replicate the positive association between emotional dominance and valence we found in Study 1.

#### Emotional dominance affects conservatism

**Fig. 4:**
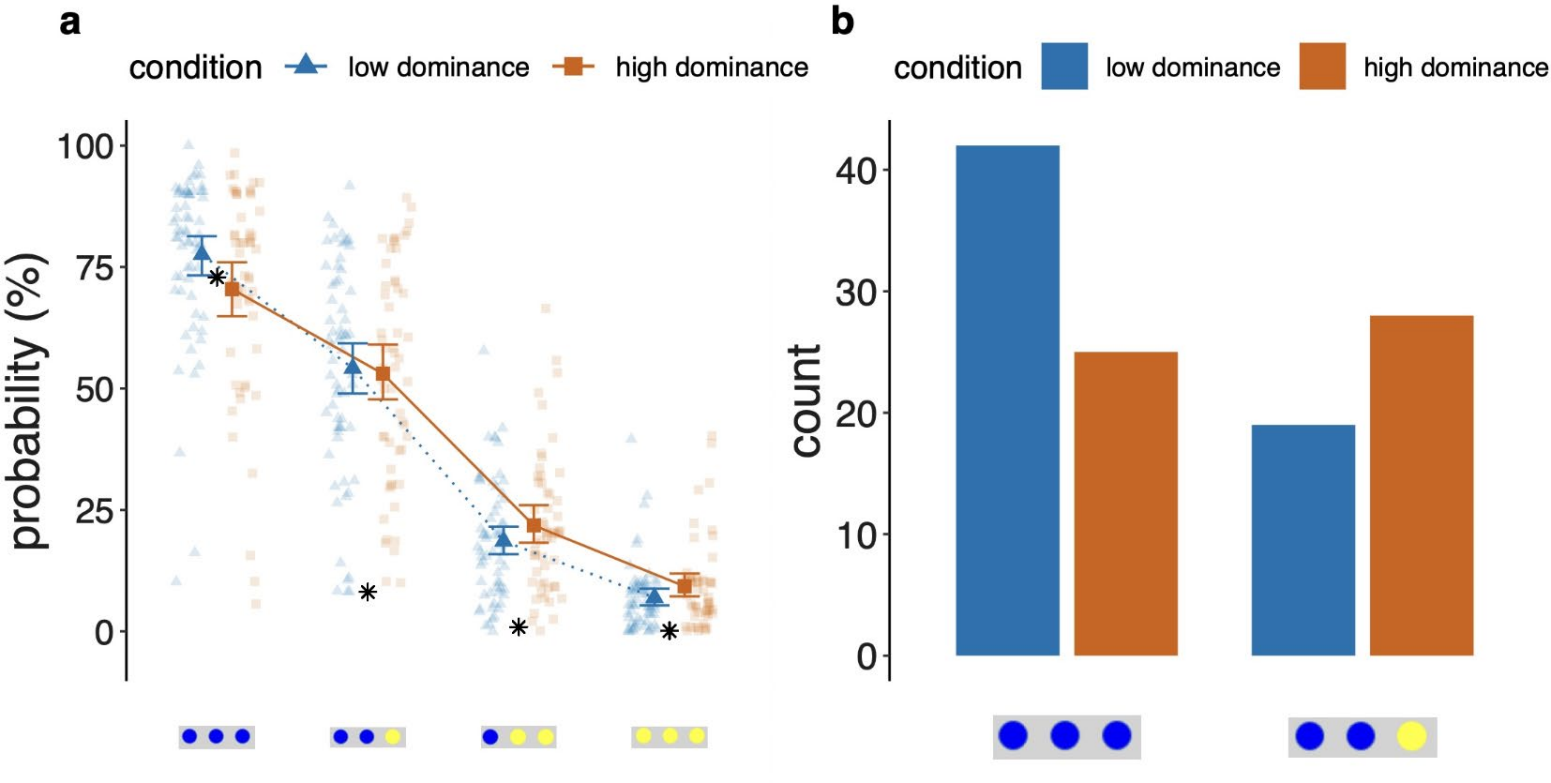
The plots show how emotional dominance affects conservatism and the use of representativeness as a proxy for mathematical probability in Study 2. (**a**) Line chart displaying probability estimates in percent for the queried compound events by experimental condition (low vs. high dominance). True probabilities are indicated as black asterisks. Mean values and 95% *CI*s are displayed for each group. (**b**) Selection of the subjectively most probable compound event by experimental condition (low vs. high dominance). Low-dominance participants had a greater rate of selecting the objectively most probable event (BBB) as most probable. High-dominance participants, in comparison to low-dominance participants, had a greater tendency to select the representative event (BBY) as most probable.

Statistical packages and analysis methods were the same as in Study 1 (see Methods section). To test the effect of the emotion induction on probability estimates, we fitted a linear mixed model predicting probability estimates by *queried compound event* and *emotion condition*. A type III ANOVA of the mixed model revealed a main effect of *queried compound event* ($\textit{F}(3, 351) = 437.73$, $\textit{p} < 0.0001$) and an interaction effect of queried compound event and emotion condition ($\textit{F}(3, 351) = 2.63$, $\textit{p} = 0.05$) on participants’ probability estimates. The results were similar for logit-transfomed values ($\textit{F}(3, 351) = 2.7$, $\textit{p} = 0.05$). Experimentally replicating findings from Study 1, we found that participants in the high dominance condition gave more similar probability estimates for the high and low probability compound events compared to participants in the low dominance condition (see Fig. 4). Bootstrapped model coefficients are listed in Table 6. Marginal $R^2$ of this model was 0.72. Compared to participants in the low dominance emotion condition, participants in the high dominance emotion condition gave lower estimates for the high probability compound event BBB and higher estimates for the low probability compound events BYY and YYY. In other words, inducing a high dominance emotional state increased participants’ tendency to show conservatism in probability estimates for affectively neutral compound probabilities.

**Table 6:** Bootstrapped model coefficients and inferential statistics of the linear mixed model predicting participants’ probability estimates in Study 2 by condition.

Emotion condition	Coefficients			Statistics	
	$\beta$	*SE*	95 % *CI*	*t*	*p*
Intercept	74.00	1.52	[71.049, 76.961]	48.76	0.000***
cond	$-$ 5.03	2.15	[$-$ 9.208, $-$ 0.848]	$-$ 2.34	0.02*
bby	$-$ 20.40	2.05	[$-$ 24.390, $-$ 16.419]	$-$ 9.98	0.000***
byy	$-$ 53.82	2.05	[$-$ 57.806, $-$ 49.835]	$-$ 26.31	0.000***
yyy	$-$ 65.89	2.05	[$-$ 69.878, $-$ 61.907]	$-$ 32.22	0.000***
cond[l]:bby	4.23	2.89	[$-$ 1.410, 9.863]	1.46	0.14
cond[l]:byy	7.32	2.89	[1.679, 12.952]	2.53	0.01*
cond[l]:yyy	6.67	2.89	[1.041, 12.313]	2.31	0.02*

As in Study 1, we next tested whether emotion condition also had an effect on probability estimates for the different compound events on an individual level. For this, we extracted regression coefficients for each participant individually (Regression Coefficient Analysis, regressing *queried compound event* on *probability estimates* as in Study 1), both for logit-transformed and raw probability estimates. We then tested whether the difference in regression coefficients between conditions was significant using the two-sided Wilcoxon signed rank test. For raw probability estimates, regression coefficients in the high dominance condition were descriptively less negative ($M_{\textit{coef}} = -17.42$, $\textit{SD} = 28.94$) than in the low dominance condition ($M_{\textit{coef}} = -23.39$, $\textit{SD} = 25.25$), but this difference was not significant ($\textit{W} = 2058$, $\textit{p} = 0.11$). For logit-transformed probability estimates (reflecting the extremeness of probability estimates), regression coefficients in the high dominance condition were significantly less negative ($M_{\textit{coef}} = -0.91$, $\textit{SD} = 1.64$) than in the low dominance condition ($M_{\textit{coef}} = -1.3$, $\textit{SD} = 1.33$, $\textit{W} = 2161$, $\textit{p} = 0.03$, $\textit{d} = 0.14$). A visualization of this result can be found in Fig. 3. In other words, in the high dominance condition, individual logit-transformed slopes were less steep and regression coefficients more centered around 0. An increase in emotional dominance made people’s probability estimates more conservative, that is, decreased the difference between estimates for high and low probability compound events.

#### Emotional dominance affects representativeness judgments

In Study 1, high dominance individuals were more likely than low dominance individuals to evaluate probabilities based on representativeness. Thus, in a subsequent step, we analyzed participants’ selection of the subjectively most probable compound event as a function of emotion condition. For this, we computed a logistic regression with *emotion condition* as predictor and participants’ choice of the subjectively most probable compound event (contrasting BBB = highest probability and BBY = highest representativeness). Participants in the low dominance condition had a higher likelihood of choosing BBB over BBY as the most probable compound event than participants in the high dominance condition (see Fig. 4, bootstrapped $\textit{B} = 0.64$, $\textit{SE B} = 0.27$, $\textit{p} = 0.02$, 95% *CI* [0.11, 1.19]; $\textit{OR} = 1.9$, 95% *CI* [1.11, 3.29]). In other words, participants in the high dominance condition had a stronger tendency to choose the more representative compound event over the mathematically most probable one. These results confirm our findings from Study 1, that is, that emotional dominance is positively associated with the use of representativeness as a proxy for probability. Our findings further imply that this relationship is causal: emotional dominance affects how people derive probabilities. Increased emotional dominance makes it more likely that a person bases her evaluations on representativeness instead of the axioms of probability.

#### Dominance-specific conservatism transfers to realistic risk assessments

**Fig. 5:**
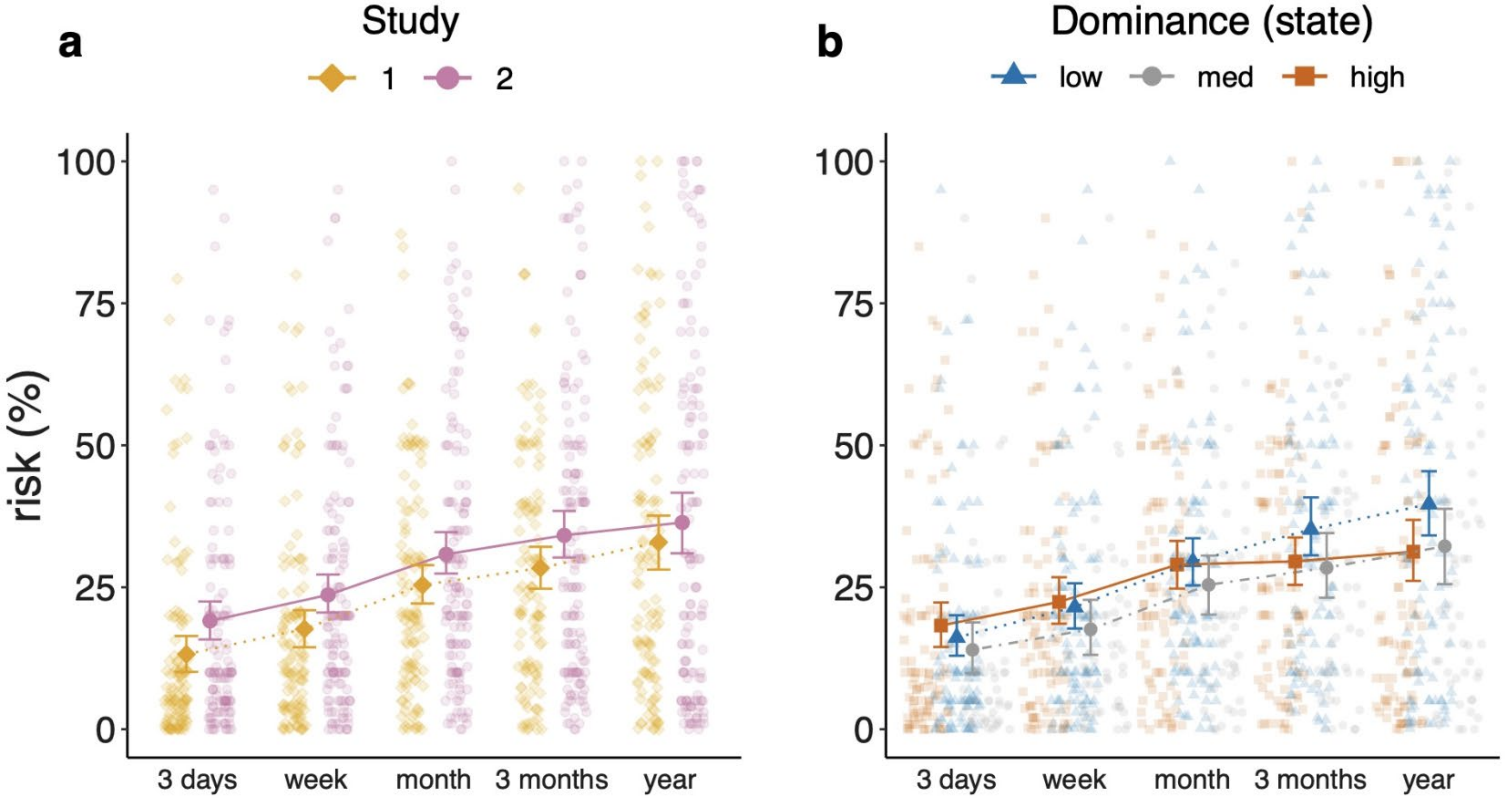
COVID-19 infection risk estimates in Studies 1 and 2 (left) and as a function of state dominance (right). (**a**) Line chart displaying prospective COVID-19 infection risk estimates at the onset of the Covid-19 pandemic (Study 1) and one year into the pandemic (Study 2). Risk estimates were generally higher at the onset of the pandemic than one year into the pandemic. (**b**) Line chart displaying COVID-19 infection risk estimates by state dominance (aggregate data from Studies 1 and Study 2). Participants high in state emotional dominance gave more optimistic long term infection risk estimates than participants low in emotional dominance, resulting in more similar short- and long term risk estimates, which can be interpreted as a form of conservatism.

Next, we investigated whether the patterns we found for dominance-specific conservatism would transfer to realistic risk assessments. We asked participants at the onset of the COVID-19 pandemic (Study 1) and one year into the pandemic (Study 2) to indicate their prospective estimates in percent for the risk of being infected with COVID-19 within the next *3 days*, *week*, *month*, *3 months*, and *year*. First, we fitted a linear mixed model with *study* as the between-subjects predictor, queried *time interval* as the within-subjects predictor, and *risk estimates* as the dependent variable, to test whether infection risk perceptions had changed over the course of the pandemic. We found significant main effects of queried *time interval* ($\textit{F}(4, 1056) = 61.81$, $\textit{p} < 0.0001$) and *study* ($\textit{F}(1, 264) = 5.58$, $\textit{p} = 0.02$). In both studies, participants gave higher risk estimates with increasing length of time interval, and in Study 2 risk estimates were generally higher (Fig. 5a). Thus, *study* was included as a control variable in subsequent analyses. Because of the shared variance between emotional dominance and valence, we also included emotional valence as a control variable in our analyses. In a next step, we analyzed the relationship between emotional dominance and conservatism in participant’s COVID-19 infection risk estimates. More specifically, we tested whether the pattern we found for dominance-specific conservatism in neutral compound probability estimates would generalize to COVID-19 risk estimates. We aggregated data from both studies and fitted a linear mixed model with *study*, *state dominance* and *state valence* as well as the interaction between these emotion variables and the within-subjects variable *time interval* as predictors and *risk estimates* as the dependent variable. In this model, the effect of *time interval* was significant ($\textit{F}(4, 1052) = 11.65$, $\textit{p} < 0.0001$), as was the effect of *study* ($\textit{F}(1, 262) = 6.27$, $\textit{p} = 0.013$). There was also a main effect of valence ($\textit{F}(1, 262) = 5.38$, $\textit{p} = 0.02$): Participants reporting lower emotional valence at the time of the study generally gave lower risk estimates for a COVID-19 infection. Furthermore, the interaction between *state dominance* and *time interval* was significant ($\textit{F}(4, 1052) = 3.67$, $\textit{p} = 0.005$). We conducted the same analysis for *trait dominance* and *valence*. In this model, neither trait *dominance* nor *valence* predicted risk estimates beyond *study* and *time interval* (all $p > 0.05$). These results reproduce the pattern we found for neutral compound probability estimates, suggesting that the modulating role of state emotional dominance in probabilistic cognition transfers to realistic contexts such as the COVID-19 pandemic. Irrespective of emotional valence, participants experiencing higher emotional dominance showed increased conservatism, giving more similar risk estimates for long (high probability) and short (low probability) time intervals. An interpretation of this finding is that emotional dominance modulates cognitive processes on a fundamental level, manifesting itself both in probability estimates of neutral compound events as well as risk estimates in realistic contexts. Alternative interpretations are discussed in the Discussion section. A visualization of these relationships can be found in Fig. 5. We also provide a more detailed analysis of participants’ risk estimates in Supplementary Analyses S2.

#### Emotional dominance as a unifying construct

Over the past decades of research on the modulating role of emotions in cognitive processes^52^, the emotion dimension dominance has received relatively little attention. This is surprising, given its usefulness for differentiating between emotions that share the same valence and arousal (e.g., anger and anxiety)^39^, its distinct neural representation^53^, and its conceptual proximity to both valence- and appraisal-centered emotion theories.

Our results suggest that emotional dominance is an important contributor to variability in probabilistic cognition. But what exactly is emotional dominance? To better understand the general cognitive and emotional patterns associated with emotional dominance, and to characterize emotional dominance in relation to leading psychological theories of emotion and cognition, we investigated the relationship between emotional dominance, the emotion dimensions valence and arousal, and appraisal of certainty and control. These data were collected in Study 1. Dominance was positively associated with valence (*p* = 0.0001, *r* = 0.29) and *control* $(\textit{p} = 0.002, \textit{r} = 0.24)$ and negatively associated with *current uncertainty* $(\textit{p} = 0.02, \textit{r} = -0.18)$ and *future uncertainty* $(\textit{p} = 0.01, \textit{r} = -0.2)$. A visualization of the correlations can be found in the correlation plot in Supplementary Figure S2; a more detailed analysis of the relationship between emotions and cognitive appraisal is provided in Supplementary Analyses S1. Previous research has found that a) emotional valence^24,29–32^ and b) appraisal of certainty and control^19,20,28^ modulate cognitive processes and affect risk estimates. So far, these strands of research have been separated. Our findings, suggesting that an increase in emotional dominance makes people more conservative in their neutral probability and realistic risk assessments and fosters the use of representativeness as a proxy for probability, support the view that emotional dominance may be a unifying concept explaining previously reported effects of emotional valence and appraisal of certainty and control on probabilistic cognition.

## Discussion

Across two studies we found evidence supporting the hypothesis that the emotion dimension dominance modulates probabilistic cognition on a fundamental level. Emotional dominance is characterized by how controlling, influential, in control, important, dominant and autonomous a person feels. Participants high in emotional dominance gave relatively low estimates for high probability and relatively high estimates for low probability compound events. In other words, the differences between estimates for the most probable and less probable events were smaller for people high in dominance. This can be viewed as a form of conservatism^11,12,54^. An increase in emotional dominance also increased participants’ tendency to select the most representative over the mathematically most probable compound event when asked to select the most probable out of a number of compound events. This result can be interpreted as an increased tendency to use representativeness as a proxy for probability. Going beyond previous work on the role of emotions in probabilistic cognition^24,25,28^, we assessed probability evaluations of participants in neutral probability estimation tasks. This allowed us to quantify the effect of emotions on probabilistic cognition without the potentially distorting influence of the desirability of probabilistic outcomes. Our results demonstrate that emotions, particularly emotional dominance, have a fundamental modulatory role in thinking, even shaping perception of affectively neutral probabilistic events.

How can we interpret our findings in light of existing theories of probabilistic cognition? One possibility, in line with the Probability Theory Plus Noise model (PT$+$N)^17^, would be that participants high in dominance experienced more noise in the probability estimation process, resulting in stronger deviations from probability theory. A potential source of this noise would be arousal; however, we did not find elevated levels of arousal in high dominance individuals. Another model that explains human probabilistic inference is the Configural Weighted Average model (CWAM^55,56^). According to this model, people infer compound probabilities by first weighting individual probabilities, with larger weights put on small probabilities, and then adding them up. Yet these weights may not be stable across participants but instead vary depending on individual differences and situational influences, such as emotional dominance.

From a Bayesian standpoint, individuals’ probability estimates are based on continuous sampling and belief updating^16,49^. An important assumption in this context is that deviations from Bayes’s rule (and from probability theory in general) stem from people’s rational adaptations to their limited computational resources: People constantly optimize their cognitive toolbox for probabilistic Bayesian inference by learning to infer^49^. Learning occurs over time and may be subject to psychological and environmental influences, giving individuals the opportunity to differentially optimize their probabilistic inference machine for distinct psychological contexts. Such adaptations may occur in different stages of the probabilistic inference mechanism: the sampling itself or the updating process may be affected. What might it mean if mental sampling were modulated by emotional dominance? In our studies, participants were confronted with a visual representation of a generating probability distribution and had to infer different compound probabilities. According to the Bayesian Sampler model^16^, in situations like this people engage in sampling from memory in order to arrive at a subjective probability estimate. If someone already feels confident, certain and in control, then investing cognitive resources into continued sampling would be wasteful and inefficient in cases where a judgment feels already good enough. In contrast, if a person lacks confidence, certainty and control, continuing mental sampling promises to pay off as it decreases feelings of uncertainty and increases feelings of subjective control.

Yet sampling may also differ qualitatively between high and low dominance individuals. For instance, different information may come to mind depending on the emotional state someone is in. In previous studies testing the effect of stimuli valence on memory encoding and retrieval, positive valence caused broadening of memory storage and retrieval^57,58^. In our tasks, more rare events (i.e., the low probability items) may have come to mind in participants in a high dominance emotional state (which was also characterized by more positive valence), resulting in an overestimation of the probability of unlikely events. This overestimation may in turn increase probability ratings for compound events containing rare elementary events and a tendency to subjectively perceive the representative compound event as most probable. A second interpretation within a Bayesian framework is that emotional dominance modulates how much attention people pay to the prior or the likelihood in the Bayesian updating process. Participants high in dominance may focus more on the prior and weight evidence sampled from memory relatively little, resulting in conservative probability estimates. Within this interpretation, high emotional dominance may provide some form of “immunity” against incoming evidence. In this view, a person high in dominance would generally assume stability of her view of the world and thus expect her beliefs to change relatively little over time.

In future research, hierarchical Bayesian modeling^56^ could be used to better understand the mechanisms causing the dominance-induced patterns we found in people’s probability estimates, comparing the predictive power of different models of dominance-specific conservatism in probabilistic inference. A challenge here would be to find the appropriate adaptations of the models to account for variations in dominance. Another approach would be to use a sequential sampling paradigm and estimate the sampling parameters of individuals high and low in dominance. Given that the PT$+$N model and the Bayesian sampler model make similar predictions in many situations, a linear regression approach^59^ appears promising in this context. To test whether high and low dominance and valence individuals differ in the relative attention paid to prior and likelihood in the Bayesian updating process, future work could directly manipulate the strength of the prior vs. the likelihood. In these studies, a more balanced gender distribution and a more diverse sample should be tested to increase the generalizability of findings. As part of this, it would also be interesting to explore the relationship between emotional dominance and probabilistic cognition by educational profiles. One question could be whether the effect of emotional dominance on probabilistic cognition holds in individuals with high mathematics proficiency.

Our finding that dominance-specific conservatism transfers to realistic COVID-19 infection risk assessments raises additional questions about the interpretation of the role of dominance in probabilistic cognition. In contrast to the probability tasks, for which objectively correct answers exist, it is impossible to know the true risk for individual participants of a future COVID-19 infection. Thus, we cannot make any statements about the accuracy of participants’ estimates for the risk of a COVID-19 infection. What we can say, however, is that participants reporting high emotional dominance gave relatively similar short and long term infection risk assessments. Although this finding resembles the pattern we found for dominance-specific conservatism in neutral probability estimates, other explanations can be thought of. Besides a general dominance-induced cognitive mechanism, a psychological explanation for this finding is that high dominance individuals might have felt less in the grip of the pandemic and had a more optimistic mindset compared to low dominance individuals. It could also be that people high in dominance had greater ability to take actual measures against an infection (e.g., working from home, rather than in a public setting with increased infection risk) compared to people reporting low dominance. Yet, it might also be the case that the general conservatism bias we found for neutral probability assessments transfers to real-life scenarios.

So far, we have mainly focused on explaining our main findings within the context of existing theories of probabilistic cognition. Yet, a number of alternative explanations exist: For example, low emotional dominance, as compared to high dominance, may be associated with increased hesitation, careful information assessment and decreased confidence in one’s assessments. This would explain why low dominance is associated with less heuristic probability assessments and increased sensitivity to differences in the probabilities of compound events. Regarding risk assessments, the same mechanism could explain more pessimistic infection risk assessment in the context of the COVID-19 pandemic in low dominance individuals, who predicted that the risk of an infection would still be increasing one year into the future. Another interpretation of our findings, both in the context of dominance-specific conservatism and the use of the representative heuristic, is that individuals with high emotional dominance were less motivated to give accurate answers in our abstract probability task than individuals with low emotional dominance. This difference in motivation would arguably have the strongest effects on probability estimates which require more probabilistic reasoning, and where the correct answer diverges most from intuitive judgments, such as the low probability compound events. More research is needed to better understand the role of emotional dominance in probability assessments in different contexts, for example, for evaluations of elementary events, conditional probabilities, and function learning.

Emotional dominance has received relatively little attention in previous research on the relationship between emotion and cognition. One reason for this might be that emotional dominance has typically been found to explain less variance in people’s emotional reactions to environmental stimuli than valence and arousal^37^. Yet, emotional dominance is key to differentiating between emotions with similar valence and arousal patterns, for instance, anger and anxiety^39^. Our results support the hypothesis that beyond sharing variance with emotional valence, emotional dominance is characterized by unique patterns of cognitive appraisal, with high emotional dominance being associated with increased levels of subjective control and certainty. We found that emotional dominance was key for predicting conservatism, both in affectively neutral probability and real-world risk assessment, as well as the use of the representativeness heuristic in people’s probability estimates. This insight is of great value given current global challenges such as climate change and the COVID-19 pandemic. Our findings also suggest that emotional dominance may be the unifying construct that explains previous research on the effect of emotional valence and cognitive appraisal on human thinking and decision making.

## Supplementary Information

Supplementary Information.

## Data Availability

The human subjects data that support the findings of this study are available in the “Open Science Framework” repository with the identifier 10.17605/OSF.IO/9CRQ6 (https://osf.io/9crq6/?view_only=a66d8a1f900849e98c9f6c84899ff141).

## Code availability

The code that support the findings of this study are available in the “Open Science Framework” repository with the identifier 10.17605/OSF.IO/9CRQ6 (https://osf.io/9crq6/?view_only=a66d8a1f900849e98c9f6c84899ff141).
