# Supplementary material for: Subjective probability is modulated by emotions
Source: Sci Rep. 2025 Mar 14;15:8895. doi: 10.1038/s41598-025-92230-2 (PMC11909219; doi:10.1038/s41598-025-92230-2)
Supplement: Supplementary file 1 — Supplementary Information. [file 41598_2025_92230_MOESM1_ESM.pdf]

Supplementary Information

Manuscript title: Subjective probability is modulated by emotions

Authors: Lara Abel*, Eric Schulz, Jonathan D. Nelson

Supplementary Figure S1

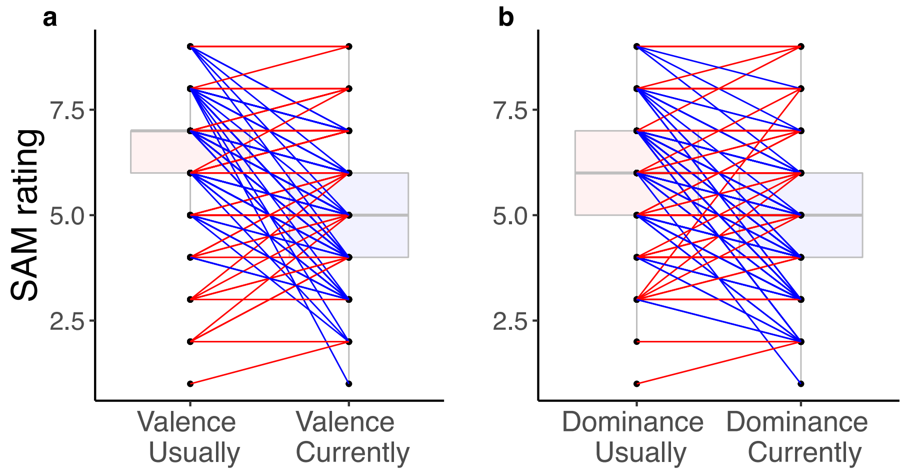

Supplementary Figure S1: *Differences between self-reported emotional dominance (**a**) and valence (**b**) before (usually experienced trait emotions, evaluated retrospectively) and at the onset of the Covid-19 pandemic in Spring 2020 (currently experienced state emotions). Ratings on the corresponding scales of the Self-Assessment-Manikin (Bradley & Lang, 1994) are plotted on the y-axis. Red lines indicate an increase, blue lines a decrease in ratings on the queried emotion dimensions.*

Supplementary Figure S2

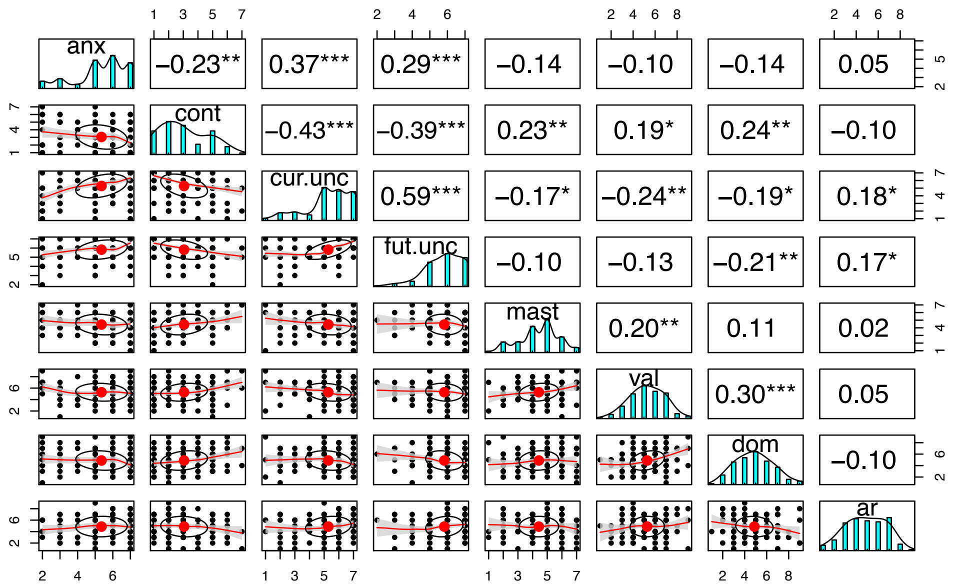

Supplementary Figure S2: *Correlation plot displaying Spearman correlations between self-reported cognitive appraisals and current (state) emotions at the onset of the COVID-19 pandemic. The center diagonal shows the histogram of participants' ratings for each of the variables: anxiety, control, current uncertainty, future-directed uncertainty, mastery, current valence, current dominance and current arousal. P-values for the Spearman correlations, plotted above the diagonal, are indicated following the convention: * indicates p ≤ 0.05, ** indicates p ≤ 0.01 and *** indicates p ≤ 0.001. The x-axis in each scatter plot below the diagonal represents the column variable, the y-axis the row variable.*

Supplementary Figure S3

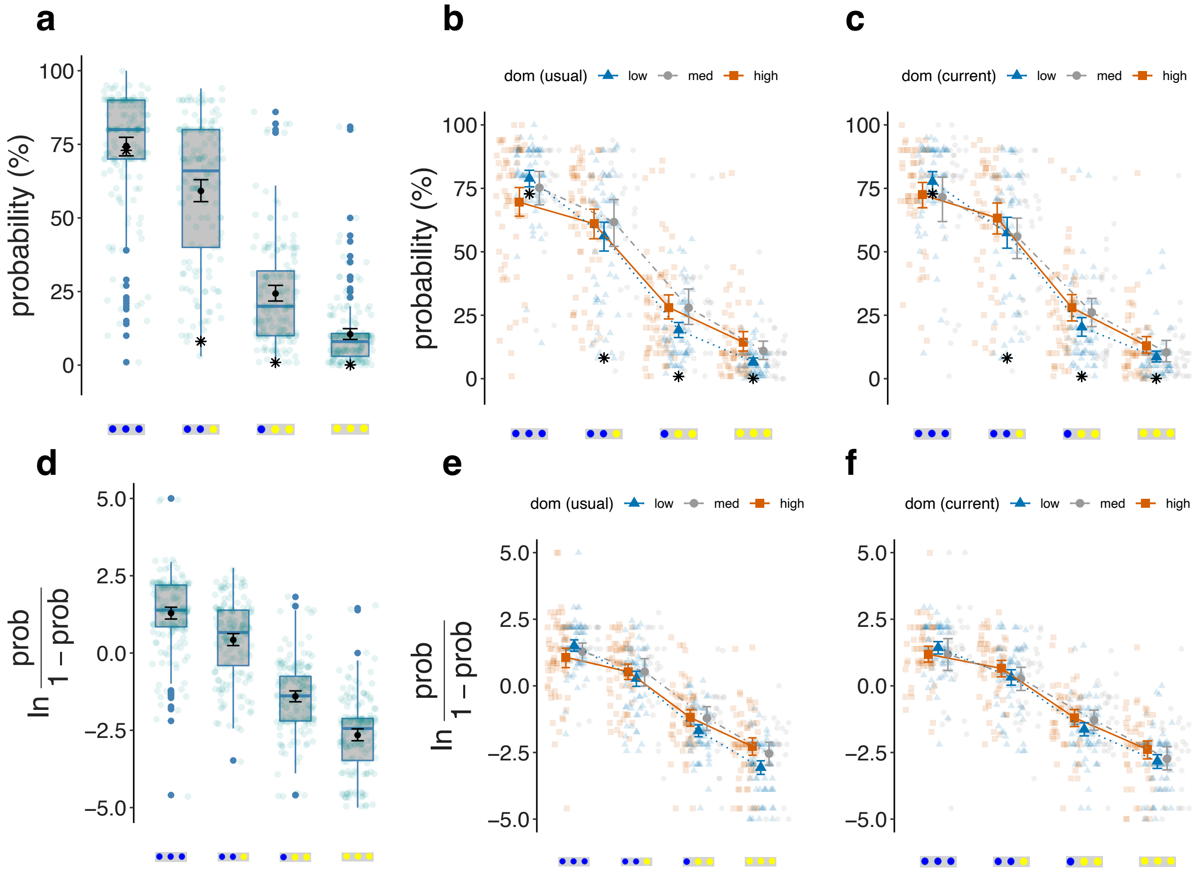

***Supplementary Figure S3**: The plots display participants' probability estimates for compound events in Task 1. Compound events for which probabilities were estimated are plotted on the x-axis. **a, b, c:** The y-axis displays probability estimates in percent. True probabilities (multiplicative model) are indicated as black asterisks. **d, e, f:** The y-axis displays log-odds transformed probability estimates. **a, d:** Box and whisker plots of participants' probability estimates. Mean values and 95% CIs are displayed in black. **b, e:** Participants' probability estimates by self-reported usual dominance before the onset of the Covid-19 pandemic (retrospectively reported trait dominance). 95% CIs are displayed for each group. Low/blue = values below the median; median/grey = values exactly at the median; high/red = values above the median. **c, f:** Participants' probability estimates by self-reported current dominance at the onset of the Covid-19 pandemic (state dominance). 95% CIs are displayed for each group. Low/blue = values below the median; median/grey = values exactly at the median; high/red = values above the median.*

Supplementary Figure S4

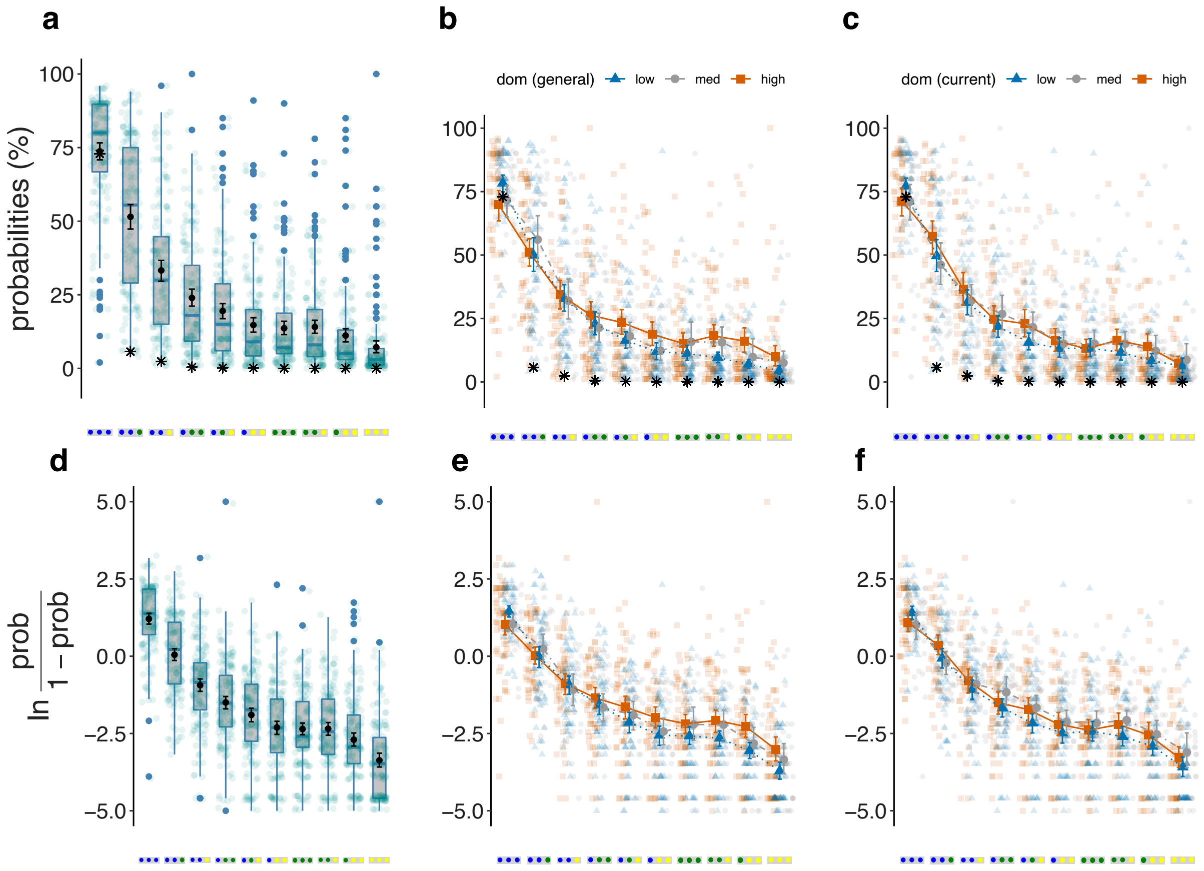

Supplementary Figure S4: *The plots display participants' probability estimates for compound events in Task 2. Compound events for which probabilities were estimated are plotted on the x-axis.* ***a, b, c:*** *The y-axis displays probability estimates in percent. True probabilities (multiplicative model) are indicated as black asterisks.* ***d, e, f:*** *The y-axis displays log-odds transformed probability estimates.* ***a, d:*** *Box and whisker plots of participants' probability estimates. Mean values and 95% CIs are displayed in black.* ***b, e:*** *Participants' probability estimates by self-reported usual dominance before the onset of the Covid-19 pandemic (retrospectively reported trait dominance). 95% CIs are displayed for each group. Low/blue = values below the median; median/grey = values exactly at the median; high/red = values above the median.* ***c, f:*** *Participants' probability estimates by self-reported current dominance at the onset of the Covid-19 pandemic (state dominance). 95% CIs are displayed for each group. Low/blue = values below the median; median/grey = values exactly at the median; high/red = values above the median.*

Supplementary Figure S5

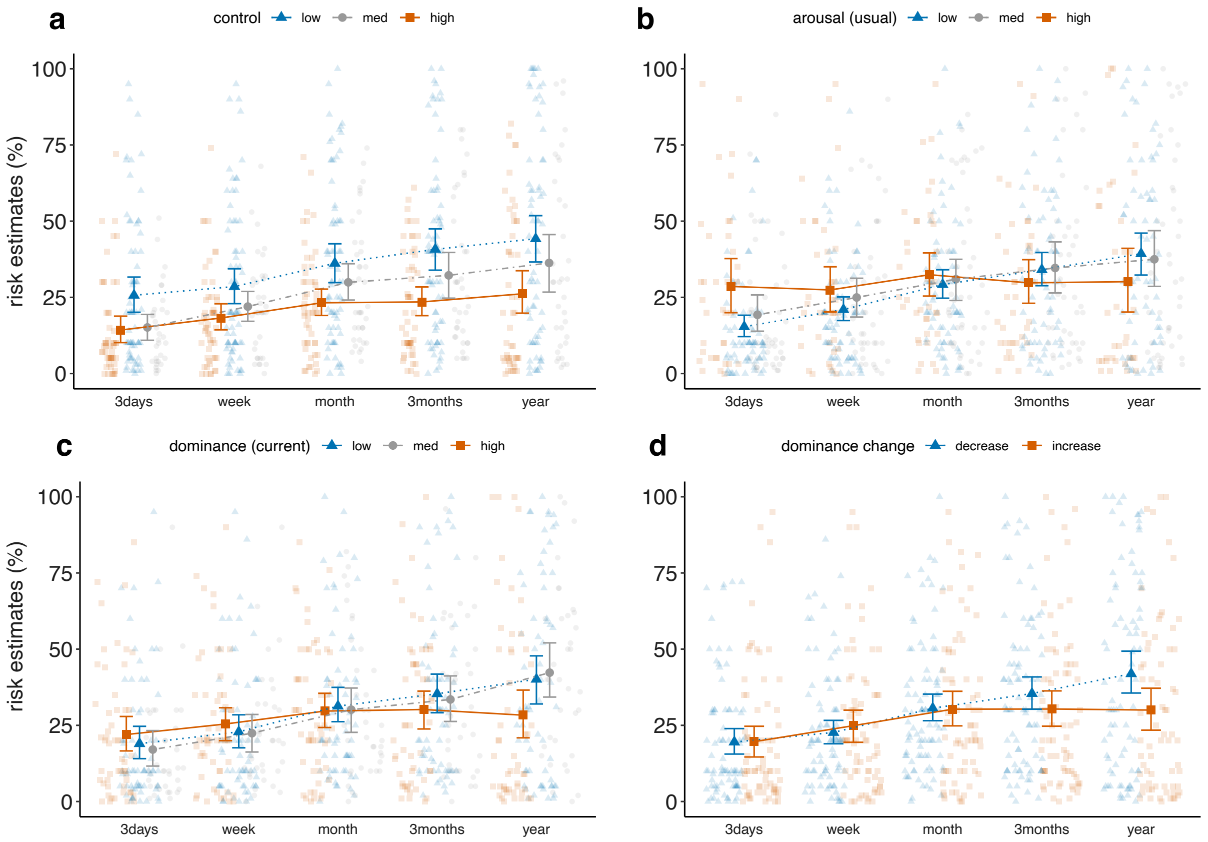

Supplementary Figure S5: *Relationship between control appraisal (**a**), trait arousal (**b**), state dominance (**c**) and dominance change (**d**) and infection risk estimates in percent at the onset of the Covid-19 pandemic. Time intervals are plotted on the x-axis and range from 3 days to one year. All data points are displayed. Mean values and 95% CIs are indicated for each group. Low/blue = values below the median; median/grey = values exactly at the median; high/red = values above the median.*

Supplementary Figure S6

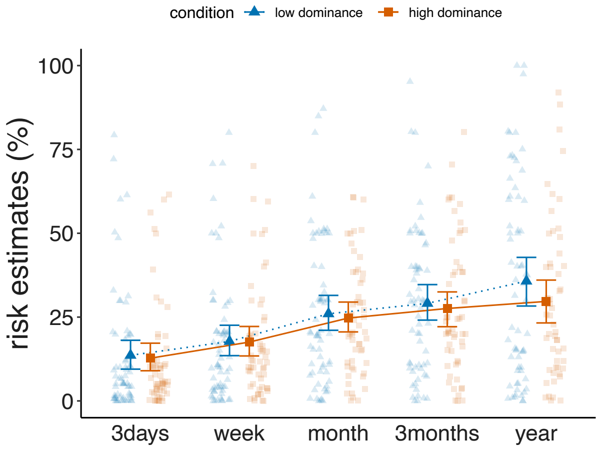

Supplementary Figure S6: *Participants' COVID-19 infection risk estimates in Study 2 (second wave of the pandemic: March 2021) by emotion condition (blue = low dominance condition, red = high dominance condition). Time intervals are plotted on the x-axis, COVID-19 infection risk estimates on the y-axis. Descriptively, participants in the high dominance condition gave more optimistic long-term estimates than participants in the low dominance condition. This interaction effect was not statistically significant (p = 0.52).*

Supplementary Figure S7

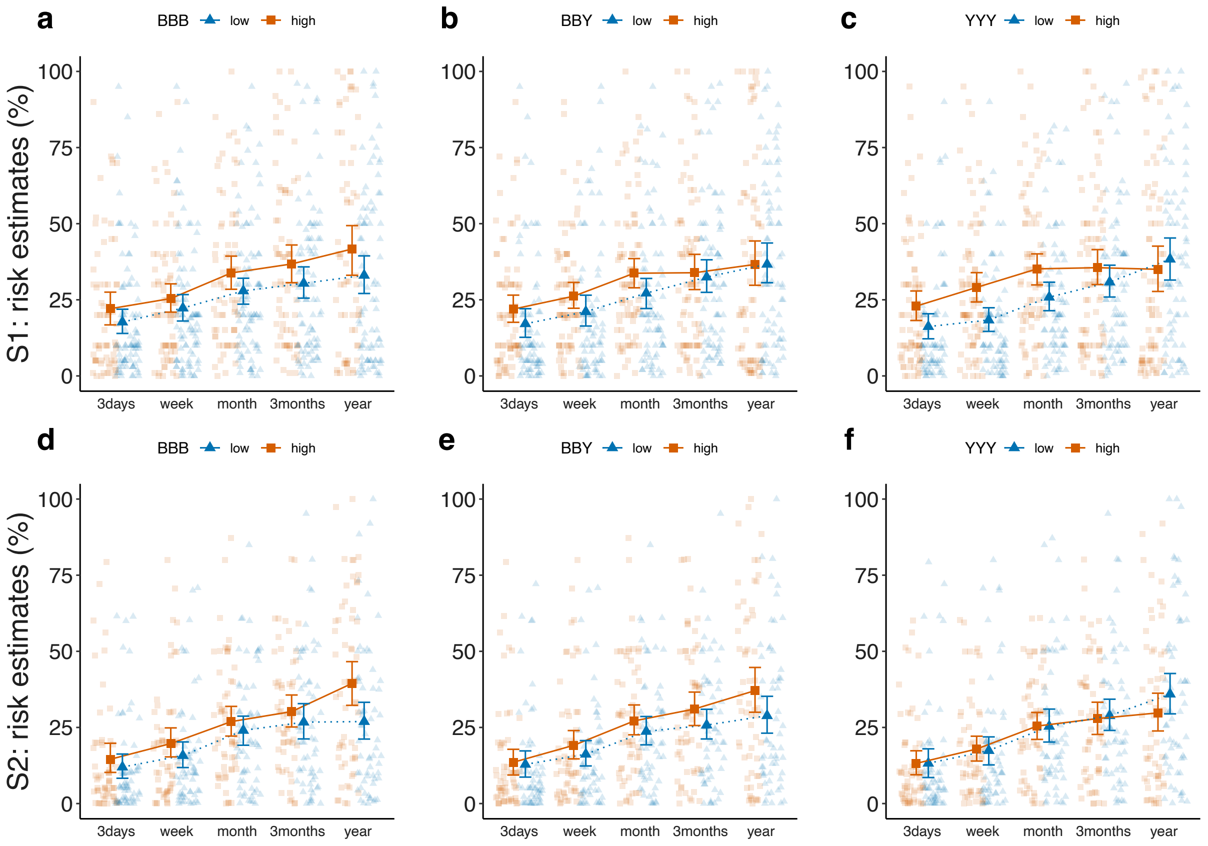

Supplementary Figure S7: *Line charts displaying the relationship between probability estimates for compound events BBB (**a, d**), BBY (**b, e**) and YYY (**c, f**) and participants' risk estimates for time intervals over the course of a year in Study 1 (**a, b, c**) and Study 2 (**d, e, f**). Participants were categorized using a median split for visualization purposes (low/blue = below median, high/red = above or equal to median). Probability estimates for the most probable compound event BBB were positively associated with overall risk estimates, and probability estimates for the less probable compound events BBY and YYY predicted the similarity between short- and long-term risk estimates (particularly in Study 1, see Supplementary Analyses 3).*

Supplementary Analyses S1:

Correlational patterns between emotions and cognitive appraisals at the onset of the COVID-19 pandemic

Emotion dimensions: Current arousal was associated with current uncertainty ($p = 0.023$, $r = 0.18$) and future uncertainty ($p = 0.02$, $r = 0.18$, ns under an adjusted α-Level of 0.01). Valence was positively correlated with mastery beliefs ($p = 0.01$, $r = 0.2$), current uncertainty ($p = 0.003$, $r = -0.23$) and control ($p = 0.02$, $r = 0.19$, ns under an adjusted α-Level of 0.01).

Cognitive appraisals and anxiety: Among the cognitive appraisals we had measured, we found significant intercorrelations between anxiety and appraisals of uncertainty regarding the current situation (current uncertainty, $p < 0.0001$, $r = 0.36$), uncertainty regarding the future (future uncertainty, $p < 0.001$, $r = 0.28$) and control ($p = 0.003$, $r = -0.23$). These findings suggest that at the beginning of the COVID-19 pandemic, people who were more anxious of an infection also experienced higher levels of uncertainty and lower levels of control regarding the pandemic situation. This finding is in line with predictions from cognitive emotion theories assuming that anxiety is associated with appraisals of high uncertainty and low control. We also found significant intercorrelations between appraisals: control was negatively associated with current uncertainty (p <

0.0001, *r* = -0.43) as well as future-oriented uncertainty ($p < 0.0001$, *r* = -0.39), meaning that participants lower in control also experienced higher levels of uncertainty regarding the current situation and the future. The belief to master challenges associated with the pandemic was negatively associated with current uncertainty (*p* = 0.05, *r* = -0.15) and positively with control (*p* = 0.004, *r* = 0.23). Notably, after adjusting the α-Level to 0.005 to account for multiple testing, the correlation between mastery and current uncertainty was not significant.

Generally, data of participants' appraisals associated with the COVID-19 pandemic showed skewed distributions: the majority of participants reported high levels of anxiety and uncertainty and low levels of control at the beginning of the pandemic.

Visualizations of the correlational relationships can be found in Supplementary Figure 2.

Supplementary Analyses S2:

Risk estimates in Study 1

We asked participants to indicate their estimated risk of a COVID-19 infection at the onset of the pandemic for prospective time intervals of three days, one week, one month, three months, and one year. To test whether participants rated their infection risk differently for different time intervals, we fitted a linear mixed model with the factor time interval as a predictor and participants as random effects. In this model, the main effect of time interval on infection risk estimates was significant: the longer the time interval, the higher participants' estimated risk of a COVID-19 infection ($p < 0.0001$). In this model, the intercept, corresponding to risk estimates for the time interval of three days, was at 19.42 (bootstrapped 95% *CI* [15.59, 23.25], *SE B* = 1.97). In this model, estimates for the intervals one week (bootstrapped *B* = 4.38, 95% *CI* [0.68, 8.07], *SE B* = 1.93), one month (bootstrapped *B* = 11.16, 95% *CI* [7.47, 14.86, *SE B* = 1.88), three months (bootstrapped *B* = 13.83, 95% CI [10.13, 17.53], *SE B* = 1.88) and one year (bootstrapped *B* = 17.26, 95% *CI* [13.51, 20.01], *SE B* = 1.93) were significantly higher than the intercept. In Tukey's post hoc tests all pairwise differences were significant (all $p < 0.05$), except for differences between three days and one week, one month and three months and three months and one year. This means that participants expected their infection risk to increase significantly if the time interval was expanded for more than one increment.

Next, we fitted separate linear mixed models with respondents' risk estimates as the dependent variable, time interval as within-subjects repeated measures variables with five levels and 1) usual valence, usual dominance and usual arousal, 2) current valence, current dominance and current arousal, 3) differences between usual and current emotions, i.e., valence change, dominance change and arousal change, and 4) anxiety and appraisals of current uncertainty, future uncertainty, control and mastery as between-subjects variables. Visualizations of significant relationships between emotions and appraisals and infection risk estimates can be found in Supplementary Figure 5.

In the first model, we found an interaction between usual arousal and time interval: Participants high in usual arousal adapted their risk estimates relatively little to the queried time interval, whereas participants medium and low in usual arousal estimated an increase in infection risk over time ($F(4, 606.78) = 4.7$, *p* = 0.001). The model's total explanatory power was 0.58 and marginal R^2 was 0.09. This model had an Intercept at 19.48 (bootstrapped 95% *CI* [15.69, 23.27], *SE B* = 1.92), corresponding to a time interval of 3 days and ratings of 0 on all three SAM scales. In this model, usual arousal modulated the difference between long-term and short-term risk assessments: participants high in usual arousal gave higher short-term risk estimates but lower long-term risk estimates than participants low or medium in usual arousal. The regression weight for usual arousal

and infection risk in the next three months was significantly negative (bootstrapped *B* = -6, 95% *CI* [-9.71, -2.29], *SE B* = 1.89), as it was for usual arousal and infection risk in the next year (bootstrapped *B* = -7.18, 95% *CI* [-10.93, -3.43], *SE B* = 1.93). Furthermore, usual emotional dominance ($F(1, 155.39) = 4.7$, $p = 0.04$) and valence ($F(1, 156.89) = 4.29$, $p = 0.04$) explained variance in people's probability estimates. Usual dominance was positively associated with the magnitude of participants' probability estimates and valence negatively. Yet, in the model the beta weights for these variables were not significant.

In the second model, current dominance interacted significantly with time interval when predicting risk estimates. Participants high in current dominance adapted their risk estimates relatively little to differences in the time interval, whereas subjects median and low in current dominance predicted an increase in infection risk over time ($F(4, 607.33) = 5.14$, $p = 0.0004$). The model's total explanatory power was 0.58 and marginal R^2 was 0.08. This model had an Intercept at 19.39 (bootstrapped 95% *CI* [15.52, 23.23], *SE B* = 1.97). Current dominance was negatively associated with risk assessments for the next year, with a significant negative beta weight for the interaction effect (bootstrapped *B* = -7.11, 95% *CI* [-11.03, -3.25], *SE B* = 1.98).

In the third model, dominance change (which was obtained by subtracting general dominance from current dominance) was a main predictor of risk estimates ($F(1, 157.73) = 4.32$, $p = 0.04$) and interacted with time interval ($F(4, 607.45) = 4.39$, $p = 0.002$). Participants reporting higher current than usual dominance (dominance increase) gave lower long-term risk estimates than subjects reporting lower current than trait dominance (dominance decrease). The model's total explanatory power was 0.58 and marginal R^2 was 0.09. This model had an Intercept at 19.39 (bootstrapped 95% *CI* [15.55, 23.20], *SE B* = 1.96). Dominance increase was associated with lower risk estimates for the time interval of one year relative to three days (bootstrapped *B* = -6.28, 95% *CI* [-10.21, -2.4], *SE B* = 1.98).

In the fourth model, control appraisal was a main predictor of risk estimates ($F(1, 155.8) = 13.23$, $p = 0.0004$). The model's total explanatory power was 0.58 and marginal R^2 was 0.14. The model had an Intercept at 19.44 (bootstrapped 95% *CI* [15.69, 23.18], *SE B* = 1.91). Participants reporting higher subjective control gave lower infection risk estimates than subjects reporting lower subjective control (bootstrapped *B* = -4.98, 95% *CI* [-9.03, -0.85], *SE B* = 2.08).

These results support the hypothesis that the emotion dimensions dominance and arousal explain variance in peoples' estimates for the risk of a COVID-19 infection at the onset of the pandemic. More specifically, self-reported usual arousal and current dominance modulated the extent to which peoples' infection risk estimates changed for different time intervals. Participants high in usual arousal gave similar estimates for all queried time intervals, adapting their estimates relatively little to differences in the referenced time intervals compared to participants medium or low in usual arousal. Participants high in current dominance gave lower long-term risk estimates than participants medium or low in current dominance but similar short-term risk estimates. This can be interpreted as increased conservatism in prospective COVID-19 infection risk assessments in participants experiencing high levels of emotional dominance.

Participants reporting high appraisals of control gave lower subjective COVID risk estimates irrespective of the referenced time interval. Interestingly, we did not find an association between emotional valence and risk estimates. This is surprising, given that previous work has found that that emotional valence modulates risk assessments. One explanation could be that in the past most research used hypothetical risk scenarios. The COVID-19 pandemic is in contrast a situation bearing a realistic risk characterized by high uncertainty. In such situations, subjective control, confidence, personal influence and autonomy, all of which are captured by the emotion dimension dominance

and are conceptually associated with appraisals of control, may be better predictors of people's risk perceptions than emotional valence.

Risk estimates in Study 2

Based on our findings from Study 1, we had hypothesized that participants in the high dominance condition would be more optimistic in their long-term COVID-19 infection risk assessments than those in the low dominance condition. To test this, we fitted a mixed linear model predicting infection risk estimates by time interval and emotion condition. A type III ANOVA of the mixed model revealed a main effect of time interval on participants' risk estimates ($F(4, 480) = 33.80$, $p < 0.0001$). In the model neither emotion condition ($p = 0.37$), nor an interaction between time interval and emotion condition ($F(4, 480) = 0.82$, $p = 0.52$) significantly affected risk estimates. Descriptively, however, long-term risk assessments were more optimistic in the high dominance condition (see Supplementary Figure 6).

Supplementary Analyses S3

Relationship between risk and probability estimates

Study 1: We fitted a linear mixed model predicting participants' prospective COVID-19 infection risk estimates by queried time interval and probability estimates and computed a type III ANOVA of the mixed model. In this model, participants' probability estimates for the compound event YYY interacted with the variable time interval when predicting COVID-19 infection risk estimates ($F(4, 601) = 4.1$, $p = 0.003$). In this model, the variable time interval did not predict infection risk estimates ($F(4, 602.2)= 0.94$, $p = 0.4$).

Study 2: We fitted a linear mixed model predicting infection risk estimates by queried time interval and probability estimates and computed a type III ANOVA of the mixed model. In this model, participants' probability estimates for the compound event BBB predicted risk estimates ($F(1, 117) = 5.86$, $p = 0.02$). In this model, the variable time interval ($F(4, 468) = 2.05$, $p = 0.09$) did not significantly predict risk estimates. Furthermore, we found an interaction between time interval and participants' probability estimates for the compound event BYY ($F(4, 468) = 3.52$, $p < 0.008$).

A visualization of the relationships between probability estimates and infection risk estimates can be found in Supplementary Figure 7.

Supplementary Analyses S4:

Psychological impact of the COVID-19 pandemic

As a proxy for the emotional impact of the COVID-19 pandemic, we asked participants to rate their *current* and retrospectively evaluated *usual* emotional valence, dominance and arousal on 9-point Self-Assessment-Manikin (Bradley & Lang, 1994) scales. Participants gave an average rating of their usual valence of $M = 6.34$. Current valence was reported at $M = 5.27$, resulting in a mean self-reported valence decrease of 1.07 points. This difference was significant in a two-sided Wilcoxon signed-rank test ($V = 1304$, $p < 0.0001$, $d = 0.39$).

On the SAM dominance scale, participants gave an average rating of their usual dominance of M = 5.85. Current dominance was at M = 4.88, a self-reported decrease of 0.97 points. This difference was significant in a two-sided Wilcoxon signed-rank test (V = 849, p < 0.0001, d = 0.36).

Current arousal (M = 4.52) and usual arousal ratings (M = 4.85) did not differ significantly (p = 0.09).

Participants subjectively evaluated their emotional valence and dominance at the onset of the COVID-19 pandemic as lower than usual (see Supplementary Figure 1). These findings match results from surveys conducted by the Kaiser Family Foundation (Kirzinger, Kearney, Hamel, & Brodie, 2020; Panchal, Kamal, Cox, & Garfield, 2021; Koma et al., 2020) reporting increased negative emotions, such as anxiety and depressive symptoms, as a psychological consequence of the pandemic. The analysis and visualization of the correlational patterns between the emotion dimensions valence, dominance and arousal, appraisals of uncertainty and control and mastery beliefs can be found in Supplementary Figure 2.

Supplementary Methods S1

COVID-19 questions

1. Subjective COVID-19 infection risk: *What do you think is your personal risk in % of being infected with COVID-19 ...*
    a. *within the next 3 days*
    b. *within the next week*
    c. *within the next month*
    d. *within the next three months*
    e. *within the next year.*

   These questions were answered before the probability task using a slider. The scale ranged from 0 to 100 (increments of 1 in Study 1, increments of 0.233/0.232 in Study 2).

2. Perceived consequences of an infection: *In case of an infection, the consequences for me personally would be ...*
   Answer options (on a 5-point Likert scale): 1 = *not even noticeable*, 2 = *noticeable*, 3 = *affecting me a bit*, 4 = *affecting me a lot*, 5 = *serious*.

3. Belonging to a risk group: *Are you in the risk group (Are you over 60 years old or do you have existing health conditions that put you in a higher risk group for COVID-19)?*
   Answer options: *Yes*; *No*; *Don't want to say*; *Not sure*

4. COVID-19 vaccination status: *Have you been vaccinated against COVID-19?*
   Answer options: *Yes, I got the first shot*; *Yes, I got both shots*; *No, I did not get vaccinated yet*

5. COVID-19 infection history: *Have you been diagnosed with Covid-19 in the past?*
   Answer options: *Yes*; *No*

6. COVID-19 related appraisal questionnaire: *Please indicate how much you agree to the following statements: Considering the current developments associated with COVID-19 ...*

    a. *I am anxious that close ones or I get infected with COVID-19*
    b. *I feel uncertain about what is going on at the moment*

c. *I feel I have control over the situation*
d. *I can master challenges associated with COVID-19*
e. *I am uncertain about the future developments associated with COVID-19*

These questions were answered on 7-point Likert scales ranging from 1 = *strongly disagree* to 7 = *strongly agree*.

Supplementary Methods S3

Emotion measure – The Self-Assessment Manikin scales

Instructions for the Valence scale were: *The following scale shows 9 different levels of emotional valence. If you select the first figure you indicate that you feel completely unhappy, annoyed, unsatisfied, melancholic, despaired, bored and if you select the last figure you indicate that you feel completely happy, pleased, satisfied, contented, hopeful. You can select any answer option between the extremes to indicate intermediate levels of emotional valence.*

Instructions for the Arousal scale were: *The following scale shows 9 different levels of emotional arousal. If you select the first figure you indicate that you feel completely relaxed, calm, sluggish, dull, sleepy, unaroused and if you select the last figure you indicate that you feel completely stimulated, excited, frenzied, jittery, wide-awake and aroused. You can select any answer option between the extremes to indicate intermediate levels of emotional arousal*.

Instructions for the Dominance scale were: *The following scale shows 9 different levels of emotional dominance. If you select the first figure you indicate that you feel completely controlled, influenced, cared for, awed, submissive, guided and if you select the last figure you indicate that you feel completely controlling, influential, in control, important, dominant, autonomous. You can select any answer option between the extremes to indicate intermediate levels of emotional dominance*.

Supplementary Methods S3

Emotion Induction

General Instructions: *In the following we ask you to think and write about three situations which could be real or imagined. Please describe these situations in 2-3 sentences each. Then we will ask you to write about one of these situations in more detail. Space for writing will be provided on the next page. Do not write about anything which may identify you or someone else. Only write about something you feel comfortable to share*.

Instructions for listing of emotional events: *Please think of a situation in which you would feel very* [...]. *Describe this situation in 2-3 sentences using the entry box below.*

Instructions for providing more detailed account of emotional event: *Please think again of the situations you just described. Please choose the situation which was emotionally most intense. Please describe in more detail (5 sentences) how you felt in that situation (e.g., physiologically, emotionally, behaviourally, cognitively) so that a person reading it would feel the same way.*

Supplementary Methods S4

Probability Task

*The following questions are **not meant as a mathematical test** but intended to better understand your **intuitions and assessment** of probability distributions.*

*Below you see a jar with **coloured balls**:*

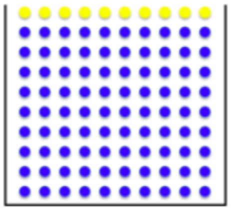

*This is an **example** of what the jar might look like after it has been mixed:*

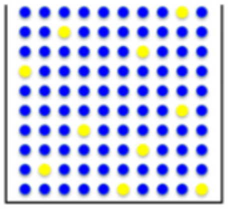

*Now we draw a **three-item-combination** from this jar: First the jar will be mixed, then a ball is drawn from the jar, its **colour is recorded** and the ball is **put back** into the jar. Then the jar will be **mixed** again, the next ball is drawn, its **colour recorded**, and put back. This procedure is **repeated three times**, resulting in a **three-item-combination**.*

What do you think, which of these colour combinations would you most likely draw?

[Additional instruction sins Study 2: *(Note that blue-blue-yellow represents drawing a blue ball in the first and second draw and a yellow ball in the third draw; blue-yellow-yellow represents drawing a blue ball in the first draw and a yellow ball in the second and third draw.)*]

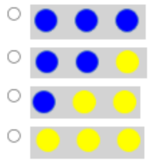

[Participants had to select exactly one of the presented compound events.]

Please indicate the probabilities in percent (0 % to 100 %) for drawing drawing these combinations.

Study 1: *(order does not matter, so blue-blue-yellow would be the same as blue-yellow-blue and yellow-blue-blue)*

Study 2: *(Again, note that blue-blue-yellow represents drawing a blue ball in the first and second draw and a yellow ball in the third draw; blue-yellow-yellow represents drawing a blue ball in the first draw and a yellow ball in the second and third draw.)*

We are interested in your intuitive answers! In case the slider is not precise enough for your estimate, please select the closest approximation.

Intuitively, how likely do you think it is it to draw...

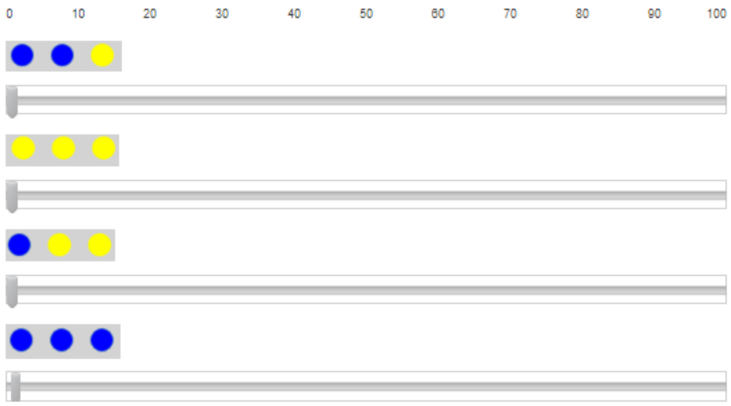

[the slider covered values ranging from 0 to 100 and could be moved with increments of 1 in Study 1 and increments of 0.235 in Study 2]
